# Artificial intelligence framework identifies candidate targets for drug repurposing in Alzheimer’s disease

**DOI:** 10.1186/s13195-021-00951-z

**Published:** 2022-01-10

**Authors:** Jiansong Fang, Pengyue Zhang, Quan Wang, Chien-Wei Chiang, Yadi Zhou, Yuan Hou, Jielin Xu, Rui Chen, Bin Zhang, Stephen J. Lewis, James B. Leverenz, Andrew A. Pieper, Bingshan Li, Lang Li, Jeffrey Cummings, Feixiong Cheng

**Affiliations:** 1 Genomic Medicine Institute, Lerner Research Institute, Cleveland Clinic, Cleveland, OH 44195 USA; 2 Department of Biostatistics and Health Data Science, School of Medicine, Indiana University, Indianapolis, IN 46202 USA; 3 Department of Molecular Physiology and Biophysics, Vanderbilt University, Nashville, TN 37212 USA; 4 Vanderbilt Genetics Institute, Vanderbilt University Medical Center, Nashville, TN 37212 USA; 5 Department of Biomedical Informatics, College of Medicine, Ohio State University, Columbus, OH 43210 USA; 6 Department of Pediatrics, Case Western Reserve University, Cleveland, Ohio 44106 USA; 7 Department of Molecular Medicine, Cleveland Clinic Lerner College of Medicine, Case Western Reserve University, Cleveland, OH 44195 USA; 8 Lou Ruvo Center for Brain Health, Neurological Institute, Cleveland Clinic, Cleveland, OH 44195 USA; 9 Harrington Discovery Institute, University Hospitals Cleveland Medical Center, Cleveland, OH 44106 USA; 10 Department of Psychiatry, Case Western Reserve University, Cleveland, OH 44106 USA; 11 Geriatric Psychiatry, GRECC, Louis Stokes Cleveland VA Medical Center, Cleveland, OH 44106 USA; 12 Institute for Transformative Molecular Medicine, School of Medicine, Case Western Reserve University, Cleveland, OH 44106 USA; 13 Department of Neuroscience, Case Western Reserve University, School of Medicine, Cleveland, OH 44106 USA; 14 Chambers-Grundy Center for Transformative Neuroscience, Department of Brain Health, School of Integrated Health Sciences, University of Nevada Las Vegas, Las Vegas, NV 89154 USA; 15 Case Comprehensive Cancer Center, Case Western Reserve University School of Medicine, Cleveland, Ohio 44106 USA

**Keywords:** Alzheimer’s disease, Drug repurposing, Genome-wide association studies (GWAS), Multi-omics, Network medicine, Pioglitazone

## Abstract

**Background:**

Genome-wide association studies (GWAS) have identified numerous susceptibility loci for Alzheimer’s disease (AD). However, utilizing GWAS and multi-omics data to identify high-confidence AD risk genes (ARGs) and druggable targets that can guide development of new therapeutics for patients suffering from AD has heretofore not been successful.

**Methods:**

To address this critical problem in the field, we have developed a network-based artificial intelligence framework that is capable of integrating multi-omics data along with human protein–protein interactome networks to accurately infer accurate drug targets impacted by GWAS-identified variants to identify new therapeutics. When applied to AD, this approach integrates GWAS findings, multi-omics data from brain samples of AD patients and AD transgenic animal models, drug-target networks, and the human protein–protein interactome, along with large-scale patient database validation and in vitro mechanistic observations in human microglia cells.

**Results:**

Through this approach, we identified 103 ARGs validated by various levels of pathobiological evidence in AD. Via network-based prediction and population-based validation, we then showed that three drugs (pioglitazone, febuxostat, and atenolol) are significantly associated with decreased risk of AD compared with matched control populations. Pioglitazone usage is significantly associated with decreased risk of AD (hazard ratio (HR) = 0.916, 95% confidence interval [CI] 0.861–0.974, *P* = 0.005) in a retrospective case-control validation. Pioglitazone is a peroxisome proliferator-activated receptor (PPAR) agonist used to treat type 2 diabetes, and propensity score matching cohort studies confirmed its association with reduced risk of AD in comparison to glipizide (HR = 0.921, 95% CI 0.862–0.984, *P* = 0.0159), an insulin secretagogue that is also used to treat type 2 diabetes. In vitro experiments showed that pioglitazone downregulated glycogen synthase kinase 3 beta (GSK3β) and cyclin-dependent kinase (CDK5) in human microglia cells, supporting a possible mechanism-of-action for its beneficial effect in AD.

**Conclusions:**

In summary, we present an integrated, network-based artificial intelligence methodology to rapidly translate GWAS findings and multi-omics data to genotype-informed therapeutic discovery in AD.

**Supplementary Information:**

The online version contains supplementary material available at 10.1186/s13195-021-00951-z.

## Background

Alzheimer’s disease (AD) is a chronic neurodegenerative disorder associated with progressive cognitive decline, extracellular amyloid plaques, intracellular neurofibrillary tangles, and neuronal death [1, 2]. AD and other dementias are an increasingly important global health burden, recently estimated to affect 43.8 million people worldwide [3]. Although genome-wide association studies (GWAS) have identified over 40 genome-wide significant susceptibility loci for AD [4–7], translating these findings into identification of high-confidence AD risk genes (ARGs) and potential therapies has eluded the field. Indeed, since Dr. Alois Alzheimer first described the condition in 1906, there are only five small-molecule drugs approved by the U.S. Food and Drug Administration (FDA) for treatment of AD: three cholinesterase inhibitors (donepezil, galantamine, and rivastigmine), one N-methyl-D-aspartate (NMDA) receptor antagonist (memantine), and one fixed combination of donepezil and memantine [8]. Aducanumab, a monoclonal antibody targeting aggregated beta-amyloid, is the first disease-modifying drug approved by U.S FDA for Alzheimer’s treatment in nearly 20 years; yet, its clinical efficacy is limited to a narrow segment of the AD continuum and potential side effects [1, 9].

The number of AD patients is expected to rise to 13.8 million by 2050 in the United States (U.S.) alone [10, 11], while the attrition rate for AD clinical trials (2002–2012) is estimated at 99.6% [12]. One possible explanation for why most candidate drugs fail in later-stage clinical trials is poor target selection. Broadly in disease, drug targets with genetic support have carried a high success rate among U.S. Food and Drug Administration (FDA)-approved therapies [13, 14]. However, this has not been the case with AD, and the translational application of multi-omics data such as GWAS for target identification and therapeutic development in AD remains challenging.

**Fig. 1:**
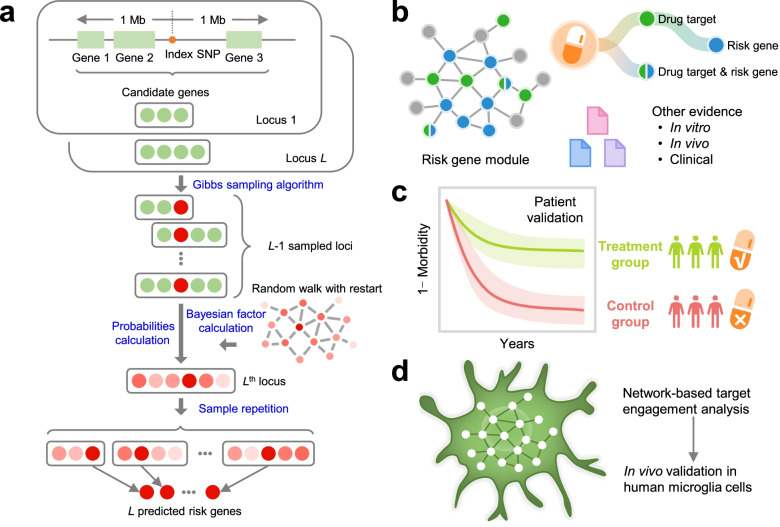
A diagram illustrating a genotype-informed, network methodology and population-based validation for Alzheimer’s therapeutic discovery. **a** A framework of network-based Bayesian algorithm (see “Material and methods”) for identifying Alzheimer’ disease (AD) risk genes. Specifically, this algorithm integrates multi-omics data and gene networks to infer risk genes from AD GWAS loci. **b** Network-based drug repurposing by incorporating ARGs and the human interactome network. **c** Population-based validation to test the drug user’s relationship with AD outcomes. Comparison analyses were conducted to evaluate the predicted drug-AD association based on individual-level longitudinal patient data and the state-of-the-art pharmacoepidemiologic methods (see “Material and methods”). **d** Network-based mechanistic observation. Experimental validation of network-predicted drug’s proposed mechanism-of-action in human microglial cells. Specifically, target prioritization and drug repurposing were conducted using network models in addition to the Bayesian algorithm. In step 1, we predicted ARGs (AD risk genes) as potential drug targets from GWAS findings using the Bayesian algorithm. In step 2, we prioritized candidate drugs via quantifying network proximity score between drug targets and ARGs under the human protein–protein interactome network models

We recently demonstrated the utility of network-based methodologies for accelerating target identification and therapeutic discovery by exploiting multi-omics profiles from individual patients in multiple complex diseases, including cardiovascular disease [15], cancer [16], schizophrenia [17], and AD [18, 19]. We now posit that systematic identification of likely causal genes by incorporating GWAS findings and multi-omics profiles with human interactome network models will also reveal disease-specific targets for genotype-informed therapeutic discovery in AD. This approach entails unique integration of the genome, transcriptome, proteome, and the human protein–protein interactome. In this study, we presented a network-based artificial intelligence (AI) framework that is capable of integrating multi-omics data along with human protein–protein interactome networks to accurately infer drug targets impacted by GWAS-identified variants to identify new therapeutics. Specifically, under the AI framework, we first apply a Bayesian algorithm to infer AD risk genes (termed ARGs) from AD GWAS loci via integrating multi-omics data and gene networks. Then repurposable drugs will be prioritized by quantifying the network proximity score [15, 16] of ARGs and drug targets in the human protein–protein interactome. Finally, we test the drug user’s relationship with AD using large-scale, longitudinal patient data and further investigate drug’s mechanism-of-action using in vitro mechanistic observations in human microglia cells (Fig. 1).

## Material and methods

### Collection of GWAS SNPs from large-scale studies

In this study, we assembled multiple single-nucleotide polymorphisms (SNPs) associated with AD from 15 large-scale GWAS studies in diverse population groups, conducted between 2007 and 2019 (Table S1). Some of the collected SNPs may represent the same genetic signal due to the use of overlapping samples across studies. To avoid this bias, we filtered the collected SNPs to remove redundant genetic signals (Supplementary Methods). To maximize genetic signals based on the omnigenic hypothesis [20], we adopted a loose threshold (*P* < 1 × 10^−5^) to collect and filter AD SNPs and finally obtained 106 unique GWAS SNPs for downstream analyses.

### Construction of human protein–protein interactome

To build a comprehensive human protein–protein interactome, we assembled data from 15 common resources with multiple levels of experimental evidence (Supplementary Methods). Specifically, we focused on high-quality protein–protein interactions (PPIs) with the following five types of experimental data: (1) binary PPIs tested by high-throughput yeast-two-hybrid (Y2H) systems; (2) kinase-substrate interactions by literature-derived low-throughput and high-throughput experiments; (3) literature-curated PPIs identified by affinity purification followed by mass spectrometry (AP-MS), Y2H, and by literature-derived low-throughput experiments, and protein three-dimensional structures; (4) signaling network by literature-derived low-throughput experiments; (5) protein complex data (see Supplementary Methods). The genes were mapped to their Entrez ID based on the National Center for Biotechnology Information (NCBI) database (www.ncbi.nlm.nih.gov), and duplicated pairs were removed. Collectively, the integrated human interactome included 351,444 PPIs connecting 17,706 unique proteins. More details are provided in our recent studies [15, 16].

### Collection of functional genomics data

We collected the distal regulatory element (DRE)-promoter links inferred from two studies. The first study was the capture Hi-C study of a lymphoblastoid cell line (GM12878) and we obtained 1,618,000 DRE-promoter links predicted from GM12878 [21]. The second dataset we used was from the Functional Annotation of the Mammalian Genome 5 (FANTOM5) project [22], in which cap analysis of gene expression (CAGE) technology was employed to infer enhancer-promoter links across multiple human tissues. We downloaded FANTOM5 data and obtained 66,899 enhancer-promoter links [22].

### Disease-associated genes

Open Targets database refers to a comprehensive platform for therapeutic target identification and validation [23]. We collected 527 AD-associated genes (Table S2) from the Open Targets database (accessed in September, 2019).

### Seed genes with experimental evidence for Alzheimer’s disease

We further collected 144 AD seed genes having either genetic, experimental, or functional evidence reported in large-scale GWAS studies, AD transgenic animal models, or human-derived samples (Table S2). These genes are involved in pathobiology of amyloidosis, tauopathy, or both, and genes characterizing other AD pathological hypotheses including neuroinflammation, vascular dementia, and other pathobiological pathways (Supplementary Methods).

### Brain-specific gene expression

We downloaded RNA-Seq data (transcripts per million, TPM) of 31 tissues from GTEx V8 release (accessed on March 31, 2020, https://www.gtexportal.org/home/). We defined those genes with counts per million (CPM) ≥ 0.5 in over 90% of samples as tissue-expressed genes and the other genes as tissue-unexpressed. To quantify the expression significance of tissue-expressed gene *i* in tissue *t*, we calculated the average expression 〈*E*(*i*)〉 and the standard deviation *δ*_*E*_(*i*) of a gene’s expression across all tissues evaluated. The significance of gene expression in tissue *t* is defined as

1 $${z}_E\left(i,t\right)=\left(E\left(i,t\right)-\left\langle E(i)\right\rangle \right)/{\delta}_E(i)$$

The details have been described in previous studies [15, 16].

### Gene expression from single-cell/nucleus transcriptomics

We collected mouse single-cell/nucleus RNA sequencing (sc/snRNA-Seq) data in 5XFAD brain samples versus controls from two recent studies (GSE98969 and GSE140511) [24, 25]. We also collected human snRNA-Seq datasets on AD patient brain tissues from two publications [26, 27]. The first set of human snRNA-Seq data contains 10 frozen post-mortem human brain tissues from both entorhinal cortex (EC) and superior frontal gyrus (SFG) regions. This dataset has been deposited in the AD knowledge portal (https://adknowledgeportal.synapse.org, Synapse ID: syn21788402). The raw data were deposited on Gene Expression Omnibus (GEO ID: GSE147528), which contains astrocytes, excitatory neurons, inhibitory neurons, and microglia cells [27]. We also assembled human snRNA-Seq data in AD cases versus controls with entorhinal cortex samples across six major brain cell types (GEO ID: GSE138852): microglia, astrocytes, neurons, oligodendrocytes, oligodendrocyte progenitor cells (OPCs), and endothelial cells [26].

The original sc/snRNA-Seq datasets were downloaded from the GEO database (www.ncbi.nlm.nih.gov/geo/), and detailed information of these datasets is provided in Table S3. The analyses were completed with Seurat (v3.1.5), scran (v1.16.0), scater (v1.16.1) packages in R with steps complied with the original literature [24–27]. Data were normalized using a scaling factor of 10,000, and all differential gene expression analyses were conducted by function *FindMarkers* in the Seurat R package with parameter test.use = “MAST.” All mouse genes were further mapped to unique human-orthologous genes using the Mouse Genome Informatics (MGI) database (Eppig *et al.*, 2017). Details of processing of sc/snRNA-seq data and quality control are provided in Supplementary Methods and our recent study [18].

### Gene expression from microarray

We collected human microarray data in AD cases versus healthy controls with human brain samples from two independent datasets (GSE29378 and GSE84422) [28, 29]. We also collected mouse microarray data from AD transgenic mouse vs. controls, including brain microglia of 5XFAD mice from 2 independent datasets (GSE65067 and GSE74615) [30, 31], and brain hippocampus of Tg4510 mice (GSE53480 and GSE57583) [32].

The original microarray datasets were obtained from Gene Expression Omnibus (https://www.ncbi.nlm.nih.gov/geo). Detailed information of these 6 GEO datasets is provided in Table S3. All raw expression data were log2 transformed, and all samples were quantile normalized together. Probe IDs in each dataset were mapped to NCBI Entrez IDs, and probes mapping to multiple genome regions or without corresponding entrez IDs were deleted. The items were imported to R statistical processing environment using a LIMMA/Bioconductor package. All the mouse genes were further transferred into unique human-orthologous genes using the MGI database [33]. Genes with threshold fold change (FC) > 1.2 were defined as exhibiting differential expression and prioritized as predicted AD risk genes.

### Bulk RNA sequencing data

We collected 2 RNA-seq datasets from brain or brain microglia of 5XFAD mice [34]. In addition, we obtained 4 RNA-seq datasets from brain microglia of Tg4510 mice across different months [M] age (2M, 4M, 6M, and 8M) [35]. Differential expression analysis was performed using DESeq [36], while threshold for significance of differential expression was set to FDR < 0.05 using Benjamini-Hochberg’s method. After mapping mouse genes to human-orthologous gene [33], we obtained 6 differentially expressed gene sets.

### Proteomic data in AD models

In total, 10 proteomic datasets were assembled from 3 types of AD transgenic mouse models in two recent publications [37, 38]. The first study performed global quantitative proteomic analysis in hAPP and hAPP-PS1 mouse models at young (3 month [M]) and old ages (12 M) [37]. We obtained four sets of DEPs (hAPP_3M, hAPP_12M, hAPP-PS1_3M, and hAPP-PS1_12M) after merging the DEPs from different brain regions. The second study performed quantitative proteomics to uncover molecular and functional signatures in the hippocampus of three types of transgenic mice [38]. Two of these mouse lines, including ADLPAPT (4M, 7M, 10M) that carry three human transgenes (APP, PSEN1, and tau) and hAPP-PS1 (4M, 7M, 10M) mouse, were used in this study. After mapping mouse genes to human-orthologous gene [33], we obtained 10 sets of DEPs.

### Enrichment analysis

Differentially expressed gene/protein (DEG/DEP) sets from multiple data sources were collected for enrichment analysis using Fisher’s exact test. This included a total of 6 bulk RNA-seq datasets and 10 proteomic datasets from 4 types of AD transgenic mouse models, including 5XFAD, Tg4510, ADLPAPT, and hAPP (see Supplementary Methods).

### AD risk gene prediction

We utilized a Bayesian model selection method, adapted from our recent work [17], to predict ARGs. Specifically, we collected at most 20 genes in the 2-Mb region centered at a GWAS index SNP as the candidates for that particular locus. Assigning *L* as the number of GWAS loci, and we then denoted a vector of genes with length *L*, each being from one of the *L* GWAS loci, as (*X*_*1*_, …, *X*_*L*_), and termed it as candidate risk gene set (CRGS). Assigning *N* to represent the biological network, we then calculated *P (X*_*1*_,*…*, *X*_*L*_*|N)* with the goal to select a CRGS with maximum posterior probability. Computationally, it is not feasible to enumerate all possible gene combinations, and we therefore adopted a Gibbs sampling algorithm to transition the problem into a single-dimensional sampling procedure. For example, when sampling the risk gene from candidates at the *L*th locus, we assumed that the risk genes at all other *L*-1 loci had been selected, and the sampling probability for a gene at the *L*th locus was computed as conditional on the *L-1* risk genes, based on its closeness to other *L-1* risk genes in the network. For each candidate gene *X*_*L*_ at the *L*th locus, we assigned *M*_*1*_ to represent the event that *X*_*L*_ is the risk gene at locus *L*, *M*_*0*_ represent the event that *X*_*L*_ is not the risk gene at locus *L*, and *X*_*-L*_ to represent all the selected risk genes in the other *L*-1 loci. The Bayesian model selection can be depicted as

2 $$\frac{P\left({M}_1|{X}_{-L},\kern0.5em N\right)}{P\left({M}_0|{X}_{-L},\kern0.5em N\right)}=\frac{P\left({M}_1\right)}{P\left({M}_0\right)}\frac{P\left({X}_{-L}|{M}_1,\kern0.5em N\right)}{P\left({X}_{-L}|{M}_0,\kern0.5em N\right)}$$

where $\frac{P\left({X}_{-L}|{M}_1,\kern0.5em N\right)}{P\left({X}_{-L}|{M}_0,\kern0.5em N\right)}$ is a Bayesian Factor (BF) measuring the closeness between *X*_*-L*_ and *X*_*L*_ in network *N* and $\frac{P\left({M}_1\right)}{P\left({M}_0\right)}$ is prior odds. The prior odds reflect the prior knowledge whether *X*_*L*_ is a risk gene or not and we assumed *P(M*_*1*_*)* = *P(M*_*0*_*)* in this study.

In regard to $\frac{P\left({X}_{-L}|{M}_1,\kern0.5em N\right)}{P\left({X}_{-L}|{M}_0,\kern0.5em N\right)}$, we adopted the random walk with restart (RWR) algorithm to calculate the BF. Starting from any node *n*_*i*_ in a predefined network *N*, the walker faces two options at each step: either moving to a direct neighbor with a probability 1 − *r* or jumping back to *n*_*i*_ with a probability *r*. The fixed parameter *r* is called the restart probability in RWR, and *r* was set as 0.3 in this study [17]. Let *W* be the adjacency matrix that decides which neighbor to be moved to, and *q*_*t*_ be the reaching probability of all nodes at step *t*. The RWR algorithm is formalized as

3 $${q}_{t+1}=\left(1-r\right)W{q}_t+r{s}_{n_i}$$

${s}_{n_i}$ is a vector with the *i*th element as 1 and 0 for others, which means th starting node is *n*_*i*_. Following the equation, *q*_*t*_ can be updated step by step until |*q*_*t* + 1_ − *q*_*t*_|^2^ < *T*_*rwr*_, where *T*_*rwr*_ is a predefined threshold. We set *T*_*rwr*_ as 1 × 10^−6^ [17]. The adjacency matrix *W* represents the distance between any two nodes in the network, and we adopted the same network and strategy in our previous work to calculate *W*. We calculated *P*(*X*_−*L*_| *M*_1_,  *N*) based on *W*. We mapped *X*_*L*_ to the rows of *W* and *X*_−*L*_ to the columns of *W*, and obtained a vector with the same length as *X*_−*L*_. The sum of the vector was calculated as *P*(*X*_−*L*_| *M*_1_,  *N*). In this study, we assumed *P*(*X*_−*L*_| *M*_0_,  *N*) to be the same for all different candidate genes. Through the Bayesian model selection equation,

4 $$\frac{P\left({M}_1|{X}_{-L},\kern0.5em N\right)}{P\left({M}_0|{X}_{-L},\kern0.5em N\right)}=\frac{P\left({M}_1\right)}{P\left({M}_0\right)}\frac{P\left({X}_{-L}|{M}_1,\kern0.5em N\right)}{P\left({X}_{-L}|{M}_0,\kern0.5em N\right)}$$

we obtained a value for each candidate genes at locus L. We used these values as sampling for Gibbs sampling to choose a risk gene for locus L. We then repeated the sampling across the remaining loci and iterated the sampling process until convergence. Specially, in each round of Gibbs sampling, we calculated the sampling frequency for each candidate gene. The frequency was compared with that of the previous round, and if the sum of squares of frequency differences across all selected genes was smaller than a predefined threshold (1 × 10^−4^ used in this study), then the sampling procedure was halted. Based on the sampling, we are able to assess the confidence of candidates being risk genes.

### Construction of drug-target network

We integrated six commonly used resources to collect high-quality physical drug-target interactions for FDA-approved drugs. We obtained biophysical drug-target interactions using reported binding affinity data: inhibition constant/potency (*K*_*i*_), dissociation constant (*K*_*d*_), median effective concentration (*EC*_*50*_), or median inhibitory concentration (IC_50_) ≤ 10 μM. First, we extracted the bioactivity data from the DrugBank database (v4.3) [39], the Therapeutic Target Database (TTD, v4.3.02) [40], and the PharmGKB database [41]. To improve data quality, we pooled only those items that satisfied the following four criteria: (i) binding affinities, including *K*_*i*_, *K*_*d*_, *IC*_*50*_, or *EC*_*50*_, ≤ 10 μM; (ii) the target protein has a unique UniProt accession number; (iii) proteins marked as “reviewed” in the UniProt database; and (iv) proteins are from Homo sapiens. Totally, we constructed a drug-target network including 15,367 physical drug-target interactions (edges), which connected 1608 FDA-approved drug nodes and 2251 unique human target nodes (Table S4).

### Description of network proximity

Given the set of disease proteins (*A*), the set of drug targets (*B*), then the closest distance *d*_*AB*_ measured by the average shortest path length of all the nodes to the other module in the human protein–protein interactome can be defined as:

5 $$\left\langle {d}_{AB}\right\rangle =\frac{1}{\left|\left|A\right|\right|+\left\Vert B\right\Vert}\left(\sum_{a\in A}{\mathit{\min}}_{b\in B}d\left(a,b\right)+\sum_{b\in B}{\mathit{\min}}_{a\in A}d\left(a,b\right)\right)$$

where *d*(*a*, *b*) denotes to the shortest path length between protein *a* and drug target *b*.

To calculate the significance of the network distance between a given drug and disease module, we constructed a reference distance distribution corresponding to the expected distance between two randomly selected groups of proteins of the same size and degree distribution as the original disease proteins and drug targets in the network. This procedure was run 1000 times. The mean $\overline{d}$ and standard deviation (*σ*_*d*_) of the reference distribution were used to caluculate a *z*-score (*z*_*d*_) by converting an observed (non-Euclidean) distance *d* to a normalized distance.

### Pharmacoepidemiologic validation

#### Patient cohort preparation

The pharmacoepidemiology study utilized the MarketScan Medicare Supplementary database from 2012 to 2017. The dataset included individual-level diagnosis codes, procedure codes, and pharmacy claims for 7.23 million U.S. older adults (i.e., age ≥ 65 to be eligible for Medicare benefits) per year, which represents approximately 14% of the 46 million retirees with Medicare benefits. Pharmacy prescriptions of pioglitazone, febuxostat, atenolol, nadolol, sotalol, and glipizide were identified by using RxNorm and National Drug Code (NDC). For a subject exposed to the aforementioned drugs, a drug episode is defined as the time between drug initiation and drug discontinuation. Specifically, drug initiation is defined as the first day of drug supply (i.e., first prescription date). Drug discontinuation is defined as the last day of drug supply (i.e., last prescription date + days of supply) accompanied by no drug supply for the next 60 days. Gaps of less than 60-day of drug supply were allowed within a drug episode. For example, the pioglitazone cohort included the first pioglitazone episode for each subject, as well as the glipizide cohort. Further, we excluded observations that started within 180 days of insurance enrollment. For the final cohorts, demographic variables including age, gender and geographical location were collected. Additionally, diagnoses of hypertension (HTN), type 2 diabetics (T2D), and coronary artery disease (CAD), defined by The International Statistical Classification of Diseases (ICD) 9/10 codes (Supplementary Methods, Table S5), before drug initiation, were collected. These variables were specifically selected to address potential confounding biases. Lastly, a control cohort was selected from patients not exposed to that drug (i.e., pioglitazone). Specifically, non-exposures were matched to the exposures (ratio 1:4) by initiation time of the drug, enrollment history, age, and gender. The geographical location, diagnoses of HTN, T2D, and CAD were collected for the control cohort as well.

### Outcome measurement

The outcome was time from drug initiation to diagnosis of AD, which was defined by using the ICD codes (Supplementary Methods). For the control cohort, the corresponding drug (i.e., pioglitazone) episode’s starting date was used as the starting time. For pioglitazone and glipizide cohorts, observations without diagnose of AD were censored at the end of drug episodes. Observations without diagnosis of AD were censored at the corresponding pioglitazone episode’s end date (Fig. S1).

### Propensity score estimation

We define Location = region of residence (i.e., North East, North Central, South, and West), T2D = type 2 diabetes, HT = hypertension, and CAD = coronary artery disease.

The propensity score of taking repurposing drug vs. a comparator drug was estimated by the following logistic regression model:

6 $$logit[Propensity\ Score] \sim Intercept+ Age+Gender+Location+T2D+HT+CAD.$$

Stratified Cox models were used to compare the AD risks. For repurposing drug vs. comparator drug or control, the analyses were stratified (*n* strata = 10) by the estimated propensity score. The propensity score adjusted Cox model is

7 $$log[Hazard]\sim Strata[log(Baseline\ Hazard) |Propensity \ Score]+1[Repurposing \ drug \ yes].$$

For repurposing drug vs. control, the analyses were stratified based on the subgroups defined by gender, T2D diagnose, HT diagnoses, and CAD diagnoses.

### Statistical analysis

Survival curves for time to AD were estimated using a Kaplan-Meier estimator. Additionally, propensity score stratified survival analysis was conducted to investigate the risk of AD between pioglitazone users and pioglitazone non-users, febuxostat users and febuxostat non-users, atenolol users and atenolol non-users, nadolol users and nadolol non-users, and sotalol users and sotalol non-users. In addition, we conducted new comparison studies to calculate the risk of AD between pioglitazone users and glipizide users. For each comparison, the propensity score of taking each drug was estimated by using a logistic regression model in which covariates included age, gender, geographical location, T2D diagnosis, HTN diagnosis, and CAD diagnoses. Furthermore, propensity score stratified Cox-proportional hazards models were used to conduct statistical inference for the hazard ratios (HR) of developing AD between cohorts.

### Experimental validation

#### Reagents

Pioglitazone was acquired from Topscience. Lipopolysaccharides (LPS) (Cat# L2880) and 3-(4,5-dimethylthiazol-2-yl)-2,5-diphenyltetrazolium bromide (MTT) were obtained from Sigma-Aldrich. Antibodies against Phospho-GSK3B-Y216 (Cat# AP0261), GSK3B (Cat# A2081), and CDK5 (Cat# A5730) were purchased from ABclonal Technology. CDK5-Phospho-Tyr15 (Cat# YP0380) was obtained from Immunoway (Plano, Texas, USA). All other reagents were purchased from Sigma-Aldrich unless otherwise specified.

#### Cell viability

Human microglia HMC3 cells were purchased from American Type Culture Collection (ATCC, Manassas, VA). Cell viability was detected by MTT method. In total, 5000 cells/well were plated in 96-well plates for 12 h, and then treated with pioglitazone for 48 h. After treatment, MTT solution was added to the cells to a final concentration of 1 mg/mL, and the mixture was allowed to incubate at 37 °C for 4 h. The supernatant was removed, and precipitates were dissolved in DMSO. Absorbance was measured at 570 nm using a Synergy H1 microplate reader (BioTek Instruments, Winooski, VT, USA).

#### Western blot analysis

HMC3 cells were pre-treated with pioglitazone (3 μM or 10 μM) and DMSO (control vehicle), and followed with 1 μg/mL LPS for 30 min. Cells were harvested, washed with cold PBS, and then lysed with RIPA Lysis Buffer with 1% Protease Inhibitor (Cat# P8340, Sigma-Aldrich). Total protein concentrations were measured using a standard BCA protein assay kit (Bio-Rad, CA, USA), according to the manufacturer’s manual. Samples were electrophoresed by sodium dodecyl sulfate-polyacrylamide gel electrophoresis (SDS-PAGE), then blotted onto a polyvinylidene difluoride (PVDF; EMD Millipore, Darmstadt, Germany) membrane. After transferring, membranes were probed with specific primary antibodies (1:1000) at 4 °C overnight. Specific protein bands were detected using a chemiluminescence reagent after hybridization with a horseradish peroxidase (HRP)-conjugated secondary antibody (1:3000).

## Results

### Pipeline of the network-based artificial intelligence methodology

We utilized a Bayesian model selection method to predict ARGs [17], based on the assumption that likely causal risk genes are more densely connected with each other in a biological network (Fig. 1a). By applying this Bayesian model to the 106 AD GWAS loci after being filtered the redundant genetic signals from original 366 SNPs (“Material and methods” and Table S1), we predicted 103 ARGs after merging the overlapping genes across several different loci (Table S6). Meanwhile, we also predicted a set of local background genes (LBGs) as a negative control for the following analyses [17]. We validated our 103 ARGs using multi-omics data, including functional genomic characteristics and transcriptomics, as well as proteomic profiles generated from diverse AD transgenic mice models and AD patient brain samples.

### Multi-omics validation of network-predicted risk genes in AD

**Fig. 2:**
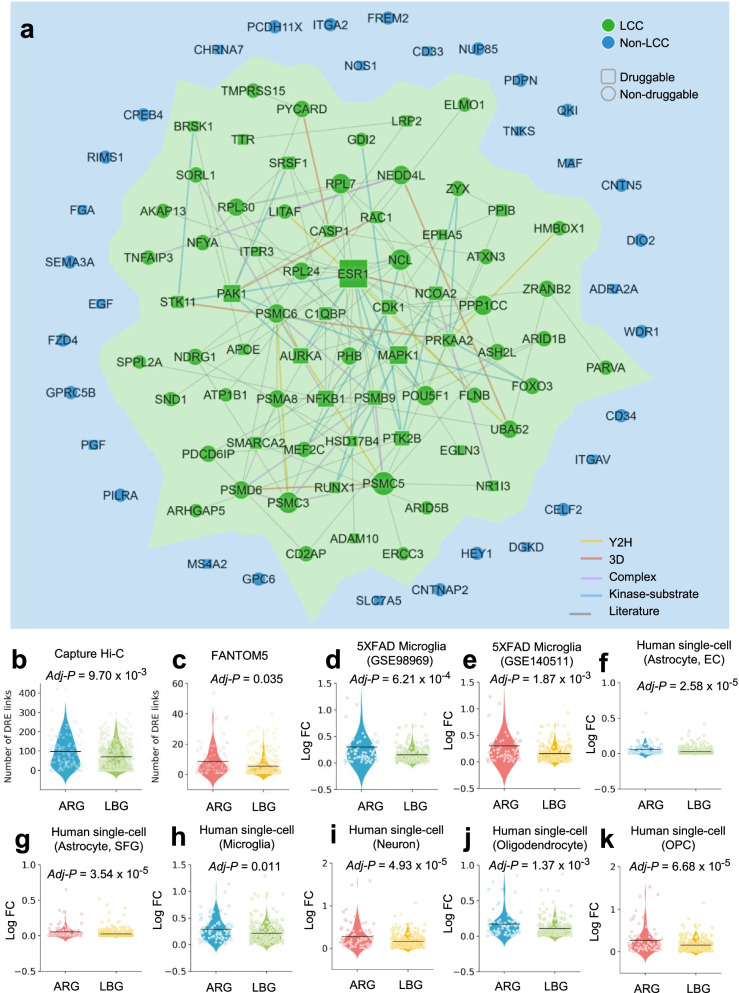
Network-based validation of predicted risk genes for Alzheimer’s disease (AD). **a** A subnetwork highlighting disease module formed by predicted AD risk genes (ARGs) in the human protein–protein interactome. This disease module includes 128 protein–protein interactions (PPIs) (edges or links) connecting 70 ARGs (nodes). Larger node size highlighting the high expression level in brain compared to other tissues. **b–k** Discovery of genomic features of 103 predicted ARGs implicated in AD. ARGs capture strong distal gene regulatory elements in Hi-C (**b**) and FANTOM5 data (**c**) compared to a set of local background genes (LBGs). **d–k** AGRs are more likely to be differentially expressed across 4 single-cell/nucleus RNA sequencing datasets (Table S3): **d, e** brain microglia cell of 5XFAD mouse model (GSE98969 [**d**] and GSE140511 [**e**]); **f,g** a human single-cell atlas (GSE147528) of entorhinal cortex (**f**) and the superior frontal gyrus (**g**) from individuals spanning the neuropathological progression of AD patient brain astrocyte cells; and a single-cell atlas (GSE138852) of entorhinal cortex from AD patients across four brain cell types: microglia [**h**], neuron [**i**], oligodendrocyte [**j**], oligodendrocyte progenitor cell (OPC) [**k**]. *P* value was computed by one-tail *T*-test. Adjusted *P* value (*adj-P*) was calculated based on the Benjamini−Hochberg approach. LCC: largest connected component; EC: entorhinal cortex; SFG: superior frontal gyrus

Recent studies showed that disease-associated proteins tend to cluster in the same neighborhood of the human protein–protein interactome, forming a disease module, a connected subgraph that contains molecular determinants of a disease [15, 16]. Disease modules are commonly used to represent the molecular determinants of disease pathobiology/physiology in a variety of human diseases, including AD [8]. We found that 103 ARGs formed significantly connected subgraphs (termed disease module) rather than being scattered randomly in the human protein–protein interactome (Supplementary Methods), consistent with previous disease module analyses that we demonstrated in other multiple complex diseases [15, 16]. Specifically, 68.0% of ARGs (70/103, *P* = 0.015, permutation test) form the largest connected subnetwork (disease module), in comparison to the same number of randomly selected genes with similar connectivity (degree) as the original seed genes in the human interactome (Fig. S2). This disease module (Fig. 2a) includes 128 PPIs (edges or links) connecting 70 unique genes (nodes). Network analysis revealed 14 genes with connectivity higher than 5, the top five of which were *ESR1*, *PSMC5*, *MAPK1*, *PAK1*, and *NFKB1*. These same five genes have previously been implicated in AD [42–45]. For example, *ESR1* interacts with tau protein in vivo, and prevents glutamate excitotoxic injury by Aβ via estrogen signaling [42]. Gene expression analysis shows that *PSMC5* was significantly overexpressed in patients carrying apolipoprotein E-ε4 (*APOE4)* mutations in comparison to *APOE* wild-type group [43]. In summary, 103 predicted ARGs comprise a strong disease module in the human interactome.

Because a majority of GWAS SNPs lie in noncoding region and exert their function by gene regulation [46], we explored the gene regulatory elements of ARGs by testing the hypothesis that the network-predicted risk genes capture more distal regulatory element (DRE)-promoter connections compared to 571 LBGs. We collected DRE-promoter connection data generated by two technologies: CAGE from FANTOM5 project and capture Hi-C (see “Material and methods”) [22, 47]. Through this, we found that the ARGs are indeed connected to more DREs in both capture Hi-C data (*adj-P* = 7.76 × 10^−3^, Fig. 2b) and FANTOM5 data (*adj*-*P* = 0.028, Fig. 2c).

We next investigated differential expression of ARGs under different pathobiology contexts of AD. We measured fold changes of gene expression levels of ARGs across 4 sc/snRNA-seq datasets (Table S3) compared to 571 LBGs. We found that ARGs were more likely to be differentially expressed in all sc/snRNA datasets (Fig. 2d–k). ARGs were more likely to be differentially expressed in (i) 5XFAD mouse brain microglial cells (Fig. 2d,e); (ii) human astrocyte cells of entorhinal cortex (Fig. 2f) and the superior frontal gyrus (Fig. 2g) from individuals spanning the neuropathological progression of AD; and (iii) a human single-cell atlas of entorhinal cortex from AD patients across four brain cell types: microglia (Fig. 2h), neuron (Fig. 2i), oligodendrocyte (Fig. 2j), and oligodendrocyte progenitor cell (OPC) (Fig. 2k). We further performed differentially expressed gene enrichment analysis for network-predicted ARGs in AD using bulk tissue expression data (Supplementary Methods). We collected bulk RNA-seq data from whole brain tissue or brain microglial cells from two common AD transgenic mouse models (5XFAD and Tg4510) and observed that ARGs were significantly differentially expressed in 5XFAD brain (*P* = 0.003), 5XFAD microglial cells (*P* = 0.002), and brain microglia of Tg4510 (Table S7). This suggests that our identified ARGs are potentially involved in diverse pathobiological pathways of AD.

We further inspected differentially expressed proteins encoded by 103 network-predicted ARGs across 10 published proteomics datasets (see Supplementary Methods) in AD. Herein, we evaluated 3 types of AD transgenic mouse models: (a) *hAPP* model containing *APP* transgene, (b) 5XFAD model harboring human transgenes for both *APP* and *PSEN1* mutations, and (c) ADLPAPT model carrying three human transgenes (*APP*, *PSEN1*, and *MAPT*). We found that products of ARGs were significantly differentially expressed in all 3 AD transgenic mouse models (*P* < 0.05, Fisher test, Table S7).

Collectively, we have thus shown that network-predicted ARGs are significantly involved in disease-related functional genomic, transcriptomic, and proteomic profiles, supporting their functional role as likely causal genes for AD.

### Incorporation of AD multi-omics data to prioritize ARGs

**Fig. 3:**
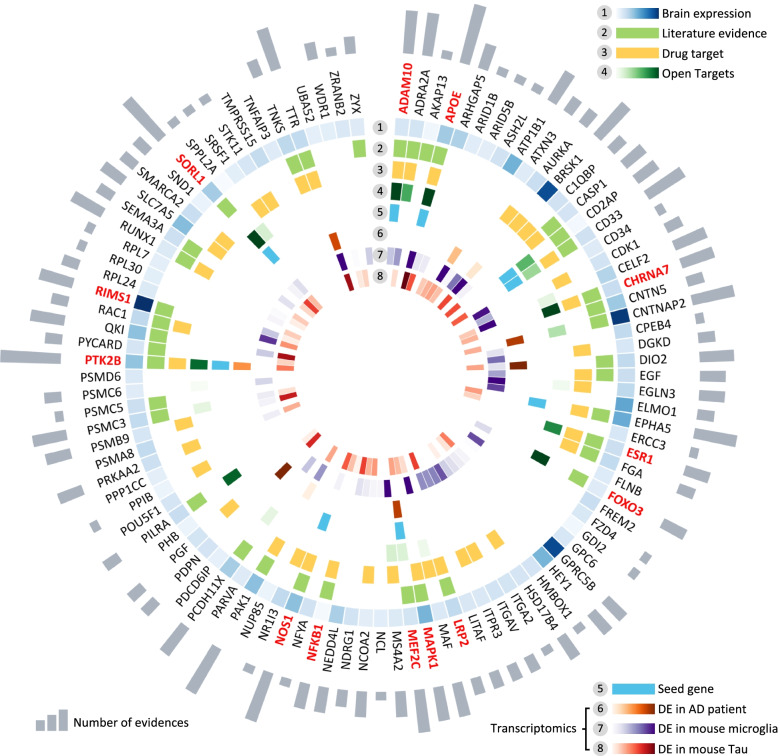
Multi-omics validation of network-predicted risk genes for Alzheimer’s disease (AD). Circle plot shows all 103 predicted AD risk genes validated by multiple-scale biological evidence. In total, 8 types of biological evidence were evaluated: (1) Brain-expression specificity derived from GTEx database (*z*-score > 0 as a high brain-specific expressed gene); (2) literature evidence validation for the gene associated with AD; (3) drug target information; (4) literature-derived experimental data from Open targets database; (5) high-quality experimentally validated AD-associated genes; (5–8) transcriptomics-based evidence (Table S3): (6) differential expression (DE) in AD patient brains; (7) differential expression in brain microglia cells of 5XFAD mouse model; (8) differential expression in brain hippocampus of Tg4510 mouse model. Gray bar denotes the number of biological evidence. A total of 13 selected risk genes involved in four AD key pathways are highlighted by red: including regulation of neurotransmitter transport, Aβ metabolic process, long-term synaptic potentiation, and oxidative stress

We next turned to prioritize high-confidence ARGs by integrating multi-omics profiles. In total, we incorporated 8 criteria that can be categorized into 5 types of biological evidence: (1) brain-expression specificity (*z*-score) derived from GTEx database, (2) availability of supportive experimental evidence from the literatures and manually curated data from Open Targets database [23], (3) experimentally validated AD genes, (4) differential gene expression from microarrays, and (5) available drug targets. Figure 3 shows a global view of 103 ARGs that we validated by these multiple forms of biological evidence in AD. Among 103 ARGs, 89 genes (86.4% [89/103]) satisfy at least one criterion. To validate the remaining 14 ARGs without any omics evidence, we further collected significantly expressed proteins or genes from the most recent human AD brain proteomic or transcriptomic studies [26, 48–51]. We found that 7 were significantly expressed in five recent human AD brain proteomics or sc/snRNA-seq datasets (Table S8). In addition, among 103 ARGs, 13 ARGs have at least 5 types of AD-related evidence, including 8 well-known AD genes: *APOE*, *PTK2B*, *NOS1*, *MEF2C*, *SORL1*, *EPHA5*, *ADAM10*, and *CD33*. For the rest of the ARGs, all but *BRSK1* had corresponding published literature-derived evidence. For example, *PAK1* is a predicted risk gene with 6 criteria of biological evidence: high brain expression specificity (*z*-score = 1.01), supportive experimental evidence from the literature, druggable target data, and differential expression in human brain of AD patients, microglial cells of 5XFAD mouse model, and brain hippocampus of a tau mouse model (Fig. 3 and Table S8). P21-activated kinase 1, encoded by the *PAK1* gene, has been implicated in AD [52], and recent studies have revealed that inactivation of *PAK1* obliterated social recognition without changing amyloid beta (Aβ)/tau pathology, and also exacerbated synaptic impairment and behavioral deficits in mouse models of AD [53, 54].

Among 103 ARGs, we selected 37 likely causal genes (Table S8) using subject matter expertise based on a combination of factors: (i) high brain-expression specificity, (ii) differential expression in multiple AD transgenic mouse models; (iii) strength of the network-based prediction, and (iv) availability of supportive experimental evidence. To advance disease understanding of network-predicted high-confidence risk genes, we performed biological pathway enrichment analysis using ClueGO plugin in Cytoscape (Table S9) [55]. We found 4 statistically significant biological pathways in AD and further discussed as below: (a) regulation of neurotransmitter transport, (b) Aβ-related biologic process, (c) long-term synaptic potentiation, and (d) oxidative stress (Table 1 and Table S10).

**Table 1:** Network-predicted risk genes involved in four pathobiological pathways of Alzheimer’s disease (AD)

Gene	Protein	Description
**Neurotransmitter transport**		
*MEF2C*^a^	Myocyte-specific enhancer factor 2C	MEF2C mRNA expression levels were correlated with AD pathology
*RIMS1*	Regulating synaptic membrane exocytosis protein 1	An altered protein expression in RIMS1 during AD pathology
**Beta-amyloid-related biologic process**		
*APOE*^a^	Apolipoprotein E	Affect Aβ production, aggregation, and clearance
*CHRNA7*	Neuronal acetylcholine receptor subunit alpha-7	Bind to Aβ with very high affinity, providing therapeutic insight into AD
*SORL1*^a^	Sortilin-related receptor	Reduce Aβ generation by trafficking APP away from amyloidogenic cleavage sites
*ADAM10*^a^	Disintegrin and metalloproteinase domain-containing protein 10	Constitutive α-secretase in the process of amyloid-β protein precursor (AβPP) cleavage
*LRP2*	Low-density lipoprotein receptor-related protein 2	rs3755166 polymorphism within LRP2 gene is associated with susceptibility to AD in the Chinese population
**Long-term synaptic potentiation**		
*MAPK1*	Mitogen-activated protein kinase 1	Beta-amyloid activates the MAPK cascade via hippocampal CHRNA7
*PTK2B*^a^	Protein-tyrosine kinase 2-beta	An in vivo modulator and early marker of Tau pathology
**Oxidative stress**		
*FOXO3*	Forkhead box protein O3	Activate BCL2L11 and FASLG to promote neuronal death and aberrant Aβ processing
*NOS1*	Nitric oxide synthase	Loss of endothelial NOS promotes p25 production and Tau phosphorylation
*NFKB1*	Nuclear factor NF-kappa-B p105 subunit	Involve in neuroinflammation, synaptic plasticity, learning, and memory implicated in AD
*ESR1*	Estrogen receptor	Interact with tau protein in vivo, and prevent glutamate excitotoxic injury by Aβ through estrogen signaling mechanisms

^a^Genes have experimental or functional evidence reported in AD transgenic animal models or human-derived samples (see Table S2 and Supplementary Method). The detailed literature data are provided in Table S2

#### Neurotransmitter transport

Specifically, *MEF2C* and *RIMS1*, encoding myocyte-specific enhancer factor 2C and regulating synaptic membrane exocytosis protein 1, play key roles in neurotransmitter secretion and synaptic plasticity. *MEF2C* (rs254776) has been reported in several AD GWAS studies [56, 57], and we found significantly lower expression of *MEF2C* in AD brain (adj-*P* = 0.010, one side Wilcoxon test, Fig. S3a). *RIMS1* is a newly predicted ARG, and a recent proteome study from human hippocampus revealed its overexpression in AD [58]. *RIMS1* is significantly overexpressed in 5XFAD mouse microglia (adj-*P* = 0.036, one side Wilcoxon test) compared to controls (Fig. S3b).

#### Beta-amyloid-related biologic process

Five genes (*APOE*, *ADAM10*, *CHRNA7*, *SORL1*, and *LRP2*) are associated with beta-amyloid biologic process. Among them, *APOE*, *ADAM10*, and *SORL1* are well-known AD risk genes, validated by large-scale genetic studies and preclinical studies [6, 59, 60]. For example, *APP* cleavage by *ADAM10* will produce an APP-derived fragment that is neuroprotective, sAPPα [61]. *CHRNA7* (neuronal acetylcholine receptor subunit alpha-7) and *LRP2* (low-density lipoprotein receptor-related protein 2) are two newly identified risk genes. There is a significantly lower expression level of *CHRNA7* in the Tg4510 mouse (adj-*P* = 3.64 × 10^−3^) compared to controls (Fig. S3e). *CHRNA7* binds to Aβ with a high affinity [62]. Finally, a previous study showed that the rs3755166 polymorphism within *LRP2* is associated with susceptibility to AD in the Chinese population [63].

#### Long-term synaptic potentiation

Mitogen-activated protein kinase (*MAPK1*) and *PTK2B* are two identified risk genes related to long-term synaptic potentiation. Mitogen-activated protein kinase 1, encoded by *MAPK1* gene, is highly expressed in brain tissue (*z*-score = 1.42). The *MAPK1* cascade can be activated by Aβ via alpha7 nicotinic acetylcholine receptors [64]. *PTK2B*, a well-known AD gene with high expression in brain (*z*-score = 0.90), was identified as an early marker and in vivo modulator of tau pathology [65], by mediating Aβ-induced synaptic dysfunction and loss [66]. There is significantly lower expression of *PTK2B* in AD patient brain transcriptome (adj-*P* = 2.05 × 10^−5^) compared to controls (Fig. S3g).

#### Oxidative stress

Oxidative stress is a prominent hypothesis in the pathogenesis of AD [67]. Here we found four network-predicted ARGs (*FOXO3*, *NOS1*, *NFKB1*, and *ESR1*) that were associated with regulation of oxidative stress. *FOXO3* encoding Forkhead box protein O3 transcription factor is a direct substrate of *CDK5*. *FOXO3* activates several genes (e.g. *BCL2L11* and *FASLG*) to promote neuronal death and aberrant Aβ processing [68]. Significantly lower expression of *FOXO3* was found in 5XFAD mouse microglia (adj-*P* = 6.90 × 10^−3^) compared to controls (Fig. S3h). *NFKB1*, encoding transcription factor nuclear factor kappa B (NF-κB), is implicated in oxidative stress, synaptic plasticity, and learning and memory [69].

Taken together, these findings suggest that our network-predicted ARGs are involved in diverse pathobiological pathways of AD. However, experimental validations are warranted for several newly predicted ARGs.

### Network-predicted ARGs are more likely to be drug targets

To date, most disease genes generated from GWAS findings are undruggable [70]. For example, a recent study revealed that none of approved and investigational AD drugs target products (proteins) of GWAS-derived genes in AD [71]. We examined whether network-predicted ARGs were more druggable compared to randomly selected proteins from human protein-coding gene background. Based on drug-target networks from 6 commonly used resources (see “Material and methods”), we obtained 2866 potential druggable proteins for FDA-approved or clinically investigational drugs. Surprisingly, we found that 41 out of 103 predicted ARGs (39.8 %) are known druggable proteins, which is four-fold higher than druggable proteins (*P* = 9.25 × 10^−11^, Fisher test) in the genome-wide human protein-coding genome background. High druggability of network-predicted ARGs offers more candidate targets for therapeutic discovery (such as drug repurposing) in AD. For example, *ADRA2A*, one of the predicted ARGs, encodes adrenoceptor alpha 2A receptor. *ADRA2A* is a potential target of clozapine [72], an atypical antipsychotic drug. Long-term clozapine treatment reduces Aβ deposition and improves cognitive impairment in an AD transgenic mice model [73]. *NR1I3*, encoding the nuclear receptor constitutive androstane receptor (CAR), is a potential drug target activated by the lipid-lowering drug simvastatin [74]. Simvastatin was reported to significantly reduce levels of Aβ in vitro and in vivo [75, 76]. In summary, network-predicted ARGs showed higher druggability compared to traditional GWAS-based analysis approaches. We next examined opportunities for drug repurposing by integrating findings from ARGs with the human protein–protein interactome network.

### ARGs offer candidate targets for Alzheimer’s drug repurposing

**Fig. 4:**
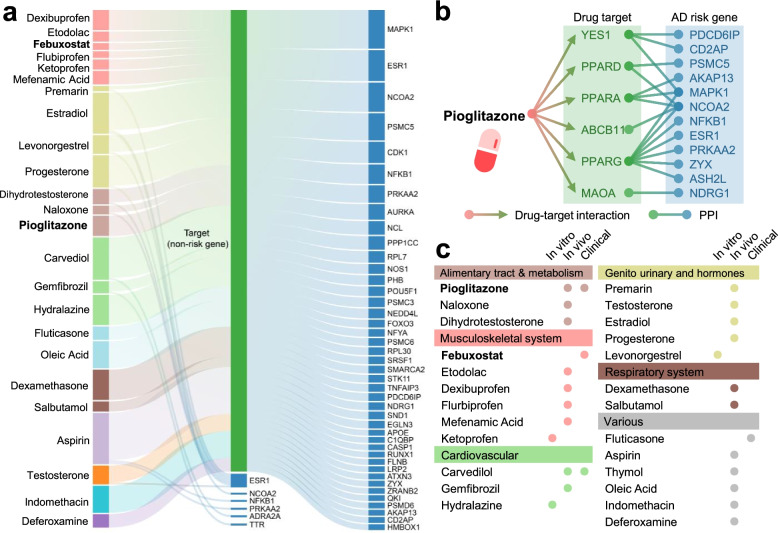
Risk gene-informed drug repurposing for Alzheimer’s disease (AD). **a** A Sankey diagram illustrates a global view of 25 repurposable drug candidates with published evidence for AD. These drugs are linked to their physical binding targets or neighborhood proteins derived from network-predicted AD risk genes. **b** Network proximity analysis measures the network distance between disease module and drug targets in the human interactome. A subnetwork indicates the molecular mechanism of pioglitazone implicated in AD, which targets six physical binding proteins of which neighborhoods are 12 predicted AD risk genes. **c** Drugs are grouped by their first-level Anatomical Therapeutic Chemical Classification (ATC) codes. The drugs with known anti-AD clinical status, in vitro and in vivo mouse model published data are given. Pioglitazone and febuxostat with anti-AD clinical evidence are highlighted

We have identified that network-predicted ARGs are related to the known pathobiology of AD and offer potential druggable targets, prompting us to examine opportunities for AD therapeutic discovery. We hypothesized that for a drug with multiple targets to be beneficial for treating a disease, its target proteins should be within or in the immediate vicinity of the corresponding disease module (Fig. 2a) in the human interactome network. To examine the potential application of ARGs on AD drug repurposing, we applied a network proximity approach [15] to quantify the interplay between AD modules from ARGs and drug targets in the human interactome network. We used the cutoff of *z*-score (*z* < − 1.5) to select network-predicted repurposable drugs in AD. After exclusion of nutraceutical drugs, metal drugs, and radioactive diagnostic agents, 130 drug candidates were obtained. We then systematically retrieved the published anti-AD clinical, in vitro/in vivo reported data for the 130 predicted drugs. In total, 25 had corresponding preclinical or clinical evidence for potential application to AD (Table S11). Figure 4 shows the molecular mechanisms of the 25 predicted drug candidates with published experimental or clinical evidence for AD. These drugs are classified into 6 categories according to Anatomical Therapeutic Chemical classification (ATC) codes: musculoskeletal systems (*n* = 6), genitourinary system and hormones (*n* = 5), cardiovascular (*n* = 3), alimentary tract and metabolism (*n* = 3), respiratory system (*n* = 2), and others (*n* = 6).

Among them, we found 4 predicted drugs having known AD clinical evidence [77, 78], including febuxostat [79], pioglitazone (NCT02913664), carvedilol (NCT01354444), and fluticasone [77]. Febuxostat, a xanthine oxidase (XO) inhibitor approved for hyperuricemia, exerts a significant network proximity (*z* = − 1.60) with the ARGs. Pioglitazone, an FDA-approved drug for T2D, has a significant network proximity (*z* = − 1.64) with the ARGs. Figure 4 shows that pioglitazone targets six proteins by connecting with 12 neighborhoods of ARGs.

### Network-predicted pioglitazone usage reduces risk of AD in patient data

**Fig. 5:**
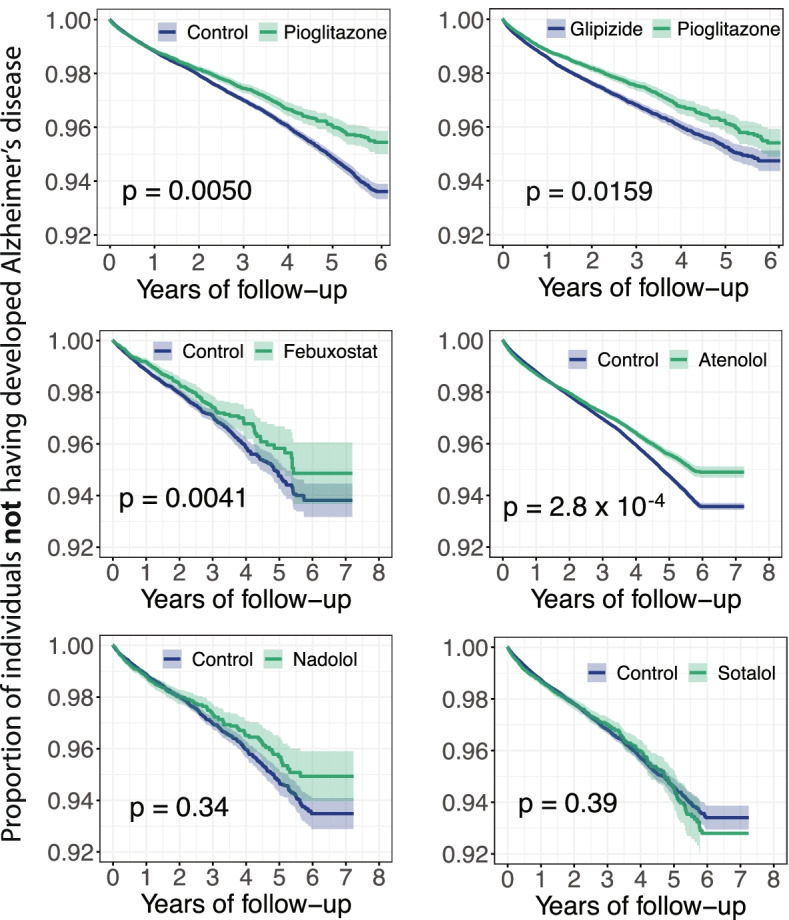
Longitudinal analyses reveal that pioglitazone reduces incidence of Alzheimer’s disease in patient data. Six comparison analyses were conducted including (i) pioglitazone (*n* = 101,650) vs. matched control population (*n* = 402,184); (ii) pioglitazone vs. glipizide (a diabetes drug, *n* = 191,656); (iii) febuxostat (*n* = 24,218) vs. control (*n* = 95,192); (iv) atenolol (*n* = 366,277) vs. control (*n* = 1,449,815); (v) nadolol (*n* = 19,253) vs. control (*n* = 76,136); and (vi) sotalol (*n* = 43,819) vs. control (*n* = 172,375). First, for each comparison, we estimated the propensity score by using the variables described in Table 2. Then, we estimated the unstratified Kaplan-Meier curves, conducted propensity score stratified (*n* strata = 10) log-rank test and Cox model. Using propensity score stratified survival analyses, non-exposures were matched to the exposures (ratio 4:1) by adjusting the initiation time of drug, enrollment history, age and gender, and disease comorbidities (hypertension, type 2 diabetes and coronary artery disease)

**Fig. 6:**
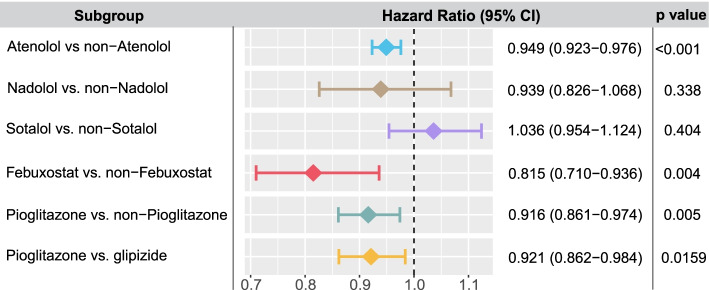
Hazard ratios and 95% confidence interval (CI) for six cohort studies. Six cohorts include the following: (i) pioglitazone (*n* = 101,650) vs. matched control population (*n* = 402,184), (ii) pioglitazone vs. glipizide (a diabetes drug, *n* = 191,656); (iii) febuxostat (*n* = 24,218) vs. control (*n* = 95,192), (iv) atenolol (*n* = 366,277) vs. control (*n* = 1,449,815); (v) nadolol (*n* = 19,253) vs. control (*n* = 76,136); and (vi) sotalol (*n* = 43,819) vs. control (*n* = 172,375). For each comparison, we estimated the propensity score for confounding factor (Table 2) adjustment as described in “Material and methods”

To test both the febuxostat and pioglitazone users’ relationships with AD outcomes using population-based validation, we conducted 2 rigorous retrospective case-control studies to compare AD risk by analyzing 7.23 million U.S. commercially insured individuals (“Material and methods”). These included the following: (i) pioglitazone (*n* = 101,650, *z* = − 1.64 [network proximity score between ARGs and drug targets in the human interactome network]) vs. a matched control population (control, *n* = 402,488), and (ii) febuxostat (*n* = 24,218, *z* = − 1.60) vs. control (*n* = 95,192). In order to identify more drug candidates with potential of reducing risk of AD, we conducted another 3 rigorous retrospective case-control studies for 3 antihypertensive agents with moderate *z*-score (*z* > − 1.0). These included the following: (iii) atenolol (*n* = 366,277, *z* = − 1.16) vs. control (*n* = 1,449,815); (iv) nadolol (*n* = 19,253, *z* = − 1.26) vs. control (*n* = 76,136); and (v) sotalol (*n* = 43,819, *z* = − 1.512) vs. a control (*n* = 172,375). Table 2 summarizes the patient data for pharmacoepidemiologic analyses. For each comparison, we estimated the unstratified Kaplan-Meier curves, conducted by both propensity score stratified (*n* strata = 10) log-rank test and Cox model. After 6 years of follow-up, pioglitazone (*P =* 0.005, hazard ratio (HR) = 0.916, 95% confidence interval [CI] 0.861–0.974), febuxostat (HR = 0.815, 95% CI 0.710–0.936, *P =* 0.004), and atenolol (HR = 0.949, 95% CI 0.923–0.976, *P* = 2.8 × 10^−4^) are associated with a reduced risk of AD compared with matched control populations (Figs. 5 and 6).

**Table 2:** Statistics of patient data used for pharmacoepidemiologic analysis

Group	Sample size	# of AD	Female (%)	Mean Age (SD)	Geographical location (%)					CAD (%)	T2D (%)	HTN (%)
					Northeast	North Central	South	West	NA			
Pioglitazone (repurposing drug), glipizide (comparative drug) and matched control												
Pioglitazone	101,650	1244	38.5	72.8 (6.9)	17.7	28.4	35.6	17.3	0.9	11.3	51.2	34.2
Glipizide	191,656	3048	44.8	74.2 (7.1)	21.5	27.6	31.4	18.7	0.8	17.1	59.4	43.0
Control	402,488	5230	38.3	74.5 (7.2)	24.2	31.4	30.5	13	1	13.5	44.6	41.1
Febuxostat (repurposing drug), and matched control												
Febuxostat	24,218	243	37.2	75.1 (7.9)	23.5	23.8	37.7	14.4	0.6	28.0	35.3	58.6
Control	95,192	1168	37.0	75.7 (7.4)	24.7	31.6	30.3	12.7	0.8	26.7	34.1	58.1
Atenolol (repurposing drug), and matched control												
Atenolol	366,277		58	74.5 (8.0)	24	26	29	21	0.7	11	14	34
Control	1,449,815		58	75.2 (7.5)	24	30	30	14	1.1	12	16	38
Nadolol (repurposing drug), and matched control												
Nadolol	19,253		60	73.7 (7.8)	29	25	35	10	0.8	11	18	34
Control	76,136		60	75.2 (7.5)	24	30	30	14	1.1	12	19	40
Sotalol (repurposing drug), and matched control												
Sotalol	43,819		46	76.4 (7.7)	19	29	38	14	0.5	35	22	50
Control	172,375		46	76.1 (7.5)	25	31	30	13	0.8	30	22	50

We estimated the unstratified Kaplan-Meier curves, conducted propensity score stratified (*n* strata = 10) log-rank test and Cox model. *CAD* coronary artery disease, *T2D* type 2 diabetes, *HTN* hypertension, *SD* standard deviation

Several clinical trials have been conducted with pioglitazone to treat AD. A phase II study (NCT00982202) showed no statistically significant difference between controls and patients with mild to moderate AD [80]. However, another study showed that pioglitazone was associated with cognitive and functional improvement, as well stabilization of AD in 42 diabetic patients [81]. Many of these studies were conducted in populations without biological confirmation of AD by biomarkers, and in some cases (e.g., the TOMMOW study; NCT01931566), the dose of pioglitazone was substantially lower than that used in clinical practice for the treatment of diabetes. The available clinical trial data do not exclude a beneficial effect of pioglitazone on AD. Except for the TOMMOW study that was conducted in cognitively normal at-risk individuals, other trials examined symptomatic patients that addressed a question different from the risk-reduction interrogation we prosecuted. For these reasons, we chose pioglitazone to conduct new comparison analysis to reduce unobserved bias.

In pharmacoepidemiologic studies, a comparator drug sharing similar indications with the investigational drug is usually selected as a “control drug” [82]. This approach is able to reduce unobserved bias, as the comparator drug and the investigational drug are likely to target the same population. Since both of pioglitazone and glipizide are treated for T2D, we therefore selected glipizide as a comparator drug. We next conducted new comparison analyses between pioglitazone and glipizide (an anti-T2D drug, *n* = 191,656) to evaluate the predicted association based on individual-level longitudinal patient data and a novel user active comparator design (“Material and methods”). New comparison analyses confirm that pioglitazone is associated with a reduced risk of AD in comparison to glipizide (HR = 0.921, 95% CI 0.862–0.984, *P* = 0.0159, Fig. 5). Thus, two independent comparison analyses support our network-based prediction for pioglitazone.

### In vitro observation of pioglitazone’s mechanism-of-action in AD

**Fig. 7:**
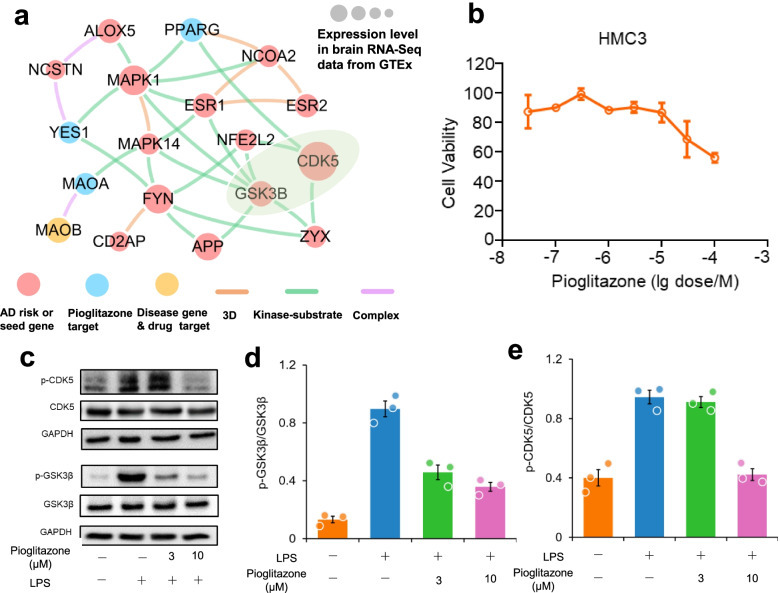
Experimental validation of pioglitazone’s proposed mechanism-of-action in Alzheimer’s disease (AD). **a** Network analysis highlighting the inferred mechanism-of-action for pioglitazone in AD. The potential molecular mechanisms of pioglitazone were inferred through integration of known drug targets and predicted AD risk or AD seed genes into brain-specific co-expressed protein–protein interactome network (see “Material and methods”). The green shadow emphasizes the two key proteins (GSK3B and CDK5) related to drug’s mechanism-of-action. Node size indicates the protein-coding gene expression level in brain compared with other 31 tissues from GTEx database (GTEx V8 release, 2020). Larger size highlighting the high expression level in brain compared with other tissues. We excluded the literature-derived protein–protein interactions. **b** Effects of pioglitazone on the cell viability of HMC3 cells. HMC3 cells were treated with indicated concentrations of pioglitazone for 48 h and cell viability was determined using MTT. Data are represented as mean ± SEM (*n* = 3) and each experiment was performed at least three times in duplicate. **c** Effects of pioglitazone on LPS-induced activation of GSK3β (**d**) and CDK5 (**e**) in human microglia HMC3 cells. HMC3 cells were pre-treated with pioglitazone and followed LPS treatment (1 μg/mL, 30 min). The total cell lysates were collected and subjected to Western blot analysis. Quantification data represent mean ± sd. of two independent experiments

Figures 5 and 6 reveal that pioglitazone significantly reduces risk of AD in longitudinal patient-based data. To further investigate its mechanism-of-action in AD, we performed a network analysis through integration of drug targets and ARGs into the brain-specific PPI network (see “Material and methods”). Network analysis shows that pioglitazone potentially targets two tauopathy-related proteins (GSK3β and CDK5) in AD (Fig. 7a). RNA sequencing data from the GTEx database (GTEx Consortium, 2015) suggests that GSK3β and CDK5 are highly expressed in brain tissue. Accumulating studies suggested that inhibition of GSK3β and CDK5 activity is a potential therapeutic strategy for AD [83].

We next examined pioglitazone’s mechanism-of-action using human microglia HMC3 cells. First, to assess the potential cell cytotoxicity, HMC3 cells were treated with pioglitazone at various concentrations (0.03 μM to 100 μM) for 48 h, and cell viability was determined by MTT method. As presented in Fig. 7b, pioglitazone at 0.03 μM to 10 μM did not affect cell viability, revealing low toxicity in human cells. Thus, these optimized concentrations of pioglitazone (≤ 10 μM) were used in subsequent experiments. As shown in Fig. 7c, phosphorylation of GSK3β and CDK5 were significantly increased after LPS treatment (1 μg/mL for 30 min) in HMC3 cells. Pre-treating with pioglitazone significantly reduced phosphorylated GSK3β and CDK5 in a dose-dependent manner (Fig. 7d,e and S4). Altogether, these data suggest that pioglitazone may offer potential benefits for patients with AD by reducing activation of GSK3β and CDK5. However, further mechanistic observations using patient-derived microglia cells or disease-relevant cell lines are warranted.

## Discussion

AD risk involves a complex polygenic and pleiotropic genetic architecture [1]. The AD genetics altered the molecular interactions of cellular pathways, which are represented through the organized structure of molecular networks (i.e., gene regulatory networks) or gene co-expression modules [84, 85]. Traditional reductionist paradigms overlook the inherent complexity of human disease and often led to treatments that are inadequate or have adverse effects [86]. Understanding AD from the point-of-view of how cellular systems and molecular interactome perturbations underlie the disease is the essence of network medicine [8]. Based on this hypothesis, we have proposed an artificial intelligence framework for AD drug repurposing, which integrates genetic findings, multi-omics data, drug-target networks, and the human protein–protein interactome, along with large-scale population-based validation and in vitro mechanistic observations in human microglia cells (see “Material and methods”).

In total, we identified 103 ARGs by utilizing our recently developed Bayesian model selection method [17]. Functional genomics enrichment analysis shows that ARGs harbor more gene regulatory elements in the human genome. Both transcriptomics and proteomics data analyses imply that ARGs are more likely to be differentially expressed in human AD brain and multiple AD transgenic mouse models. The marginal difference of fold change (Fig. 2) may be explained by large number of cell subpopulations and low abundance of RNA expression at single-cell/nucleus levels during differential expression analysis. These comprehensive observations suggest that ARGs potentially capture pathobiological pathways of AD (Fig. 3). Importantly, drug-target network analysis shows a 4-fold higher druggability compared to the known drug targets in the human genome. A previous study showed that few products (proteins) of GWAS-derived closest genes could be applied for therapeutic discovery [71].

To compare the performance between the nearest genes to the risk loci using traditional approach and ARGs derived from our Bayesian model, we assembled 108 nearest genes (Table S1) to the GWAS loci data we used (see Supplementary Methods). Unlike to 103 ARGs (Fig. 2a), 108 GWAS-derived closest genes were randomly distributed in the human interactome network (7/96, *P* = 0.217, permutation test, Table S12). In addition, among 25 candidate drugs (with known AD evidence) identified by ARGs, only 2 drugs (hydralazine and deferoxamine) exerted significant network proximity score with AD (Table S11) based on the 108 nearest genes. None of three positive drugs (pioglitazone [*z* = − 1.64], febuxostat [*z* = − 1.6], and atenolol [*z* = − 1.16]) in Fig. 5 show significant network proximity score with the 108 nearest genes. Altogether, these observations implied poor performance of GWAS-derived closest genes as candidate targets for therapeutic discovery, consistent with previous studies [17, 87]. Several factors may account for this. First, the reported significant loci occupy only a small proportion of heritability and provide limited information about underlying AD biology [88]. Second, many genome-wide significant loci lie in noncoding regions, and genes closest to index SNPs may not represent causal genes of AD [89]. Thus, systematic identification of likely causal genes from GWAS findings using in silico multi-omics approaches is a crucial step for understanding AD pathobiology and offers potential candidate targets for new therapeutic development as presented in this study.

Network-based drug repurposing from ARG findings prioritize 4 repurposable drug candidates for AD, including pioglitazone (NCT02913664), carvedilol (NCT01354444), febuxostat, and fluticasone. Carvedilol, an FDA-approved drug for hypertension that blocks the beta adrenergic receptor, significantly attenuates brain oligomeric β-amyloid level and cognitive deterioration in two independent AD mice models [90]. Fluticasone is an approved glucocorticoid receptor agonist for treatment of asthma, and recent studies showed that long-term use of fluticasone reduces incidence of developing AD [18, 77]. A propensity-matched analysis has suggested that a daily dose of 40 mg febuxostat is associated with reduced likelihood of dementia in older adults [79]. Nevertheless, whether febuxostat reduces the risk of AD dementia remains unknown. Combining network-based prediction and patient data observation, we found that febuxostat is significantly associated with a decreased risk of AD (Figs. 5 and 6). In addition, epidemiological studies have shown that hypertension is a risk factor for AD-related dementia; yet, there is some dispute as to whether antihypertensive drugs reduce the risk of AD [91, 92]. Among three adrenergic beta blocker-based antihypertensive drugs (atenolol, nadolol, and sotalol), atenolol is associated with reduced risk of AD, while nadolol and sotalol are not (Figs. 5 and 6). Since there are lack of strong preclinical or clinical evidence to support the relationship between atenolol and AD, we excluded atenolol in our follow-up studies. However, future studies to confirm potential beneficial effects of antihypertensive drugs in reducing AD risk are needed.

Pioglitazone, a U.S. FDA-approved anti-T2D drug, was reported to restore energy metabolism and reduce Aβ levels in the brain of APP/PS1 mice [93]. A previous clinical study has shown that pioglitazone improves cognition and regional cerebral blood flow in patients with mild AD accompanied with T2D [81]. In this study, by combining network-based prediction and population-based validation, we found that pioglitazone potentially reduced risk of AD in large-scale patient database (Figs. 5 and 6). Under active drug user design framework [15], we chose a comparator drug having the similar indication with the target drug pioglitazone. As pioglitazone was approved for anti-diabetes, and we therefore chose glipizide as a comparator drug. New active drug user design analysis further support that pioglitazone is associated with a reduced risk of AD in comparison to glipizide. In addition, in vitro mechanistic observations (Fig. 7) reveal that pioglitazone significantly downregulates expression of CDK5 and GSK3β in human microglia cells, mechanistically supporting network and population-based findings. However, a phase II study (NCT00982202) shows no statistically significant differences between controls and patients with mild to moderate AD for pioglitazone [80]. One possible explanation is that pioglitazone reduces risk of AD only in patients with pre-existing diabetes or that pioglitazone may have its effects before symptoms occur but not in more advanced patients. Thus, our findings suggest that larger clinical trials and additional mechanistic studies may be necessary to clarify pioglitazone’s action in AD prevention in both a broad population and a well-defined subpopulation.

Our network methodology presented here has several strengths. First, it contributes to identification of high-confidence likely causal genes, followed by multi-omics data validation, network-based drug repurposing investigation, large-scale patient data analysis, and in vitro mechanistic observation in human microglial cells. This work illustrates translation of GWAS findings to pathobiology and therapeutic development in AD. Second, our proposed network proximity approach outperform other network approaches, such as weighted gene co-expression network analysis (WGCNA) in which it infers gene co-expression networks from gene/protein expression profiles using network community detection theory [94]. Multiple studies have demonstrated high false positive rate of the gene/protein co-expression networks compared to the physical protein–protein interaction network [95, 96]. Third, the large patient-level longitudinal data ensures that our analyses integrate real-world patient evidence to test the drug’s efficiency in AD risk reduction.

### Limitations

Potential weaknesses of this work should be acknowledged. First, as genetic variants from GWAS that influence human disease traits are far from complete, a relative loose threshold (1 × 10^−5^) rather than genome-wide significant threshold (5 × 10^−8^) is adopted, which may affect the accuracy of identification of ARGs. Second, we only integrated SNPs associated with AD from large-scale GWAS studies conducted between 2007 and 2019. Since several recent GWAS studies have been conducted [97, 98], we may identify new AD-associated risk genes via integration of the latest novel GWAS loci for AD in the future. Third, incompleteness of human protein–protein interactome network data and potential literature bias may influence performance of our methodology as discussed in a recent study [99]. Since the likely causal genes were predicted based on SNPs identified from GWAS that are primarily centered on the variants in the noncoding regions, some AD genes or proteins harboring protein-coding variants may not be covered in this study. Integration of large-scale whole-genome/exome sequencing from the Alzheimer’s Disease Sequencing Project (ADSP; https://www.niagads.org/adsp/content/home) may offer novel risk genes for AD. Fourth, detailed clinical information is missing for health insurance claims data regardless of high-dimensional covariate adjustment. This limits our ability to test the effects of pioglitazone on subpopulation of AD patients such as those with mild AD. Longitudinal analyses were conducted in populations without biological confirmation of AD by biomarkers, such as lack of cerebrospinal fluid information (i.e., levels of Aβ and Tau), which may affect the results of pharmacoepidemiologic analyses. In addition, although our dataset contains a geographically diverse population of commercially insured Americans seniors, the results are not representative of individuals who are not commercially insured or uninsured. The phenotyping algorithms may not capture all AD cases. Thus, this approach may need to be re-applied on a regular iterative basis as datasets are expanded, in order to offer maximum utility. Furthermore, clinical data heterogeneity from real-world patients and other confounding factors may lead to potential false positive rate in population-based drug outcome analysis. Finally, all novel ARGs need to be validated experimentally (including both in vitro and in vivo) and clinical benefits of drugs must be tested in AD randomized clinical trials in the future.

## Conclusion

In summary, this study presents a network-based artificial intelligence methodology to translate GWAS findings to emerging therapeutic discovery by incorporating multi-omics, drug-target network, and the human protein–protein interactome, along with large-scale population-based and in vitro mechanistic observation. This study shows the strong proof-of-concept application of high-confidence risk gene identification from human genetic and multi-omics findings to identifying treatments that can be repurposed for AD and has identified pioglitazone as a potential new treatment for AD using artificial intelligence approaches. In this way, we can minimize the translational gap between genetic findings and clinical outcomes, which is a significant problem in current AD therapeutic development. From a translational perspective, if broadly applied, the artificial intelligence-based tools developed here could help develop novel efficacious treatment strategies for other human complex diseases.

## Supplementary Information

**Additional file 1.**

## Data Availability

Supplementary Material and Methods, 4 Supplementary Figures and 11 Supplementary Tables are freely available at https://github.com/ChengF-Lab/alzRG. In addition, all multi-omics data, human protein–protein interactome network, and drug-target network used this study are freely available at https://alzgps.lerner.ccf.org as well.
