# Supplementary material for: Artificial intelligence framework identifies candidate targets for drug repurposing in Alzheimer’s disease
Source: Alzheimers Res Ther. 2022 Jan 10;14:7. doi: 10.1186/s13195-021-00951-z (PMC8751379; doi:10.1186/s13195-021-00951-z)
Supplement: Supplementary file 1 — Additional file 1. [file 13195_2021_951_MOESM1_ESM.zip › SI_R1_CHENG.pdf]

Supporting Information

Title: Artificial intelligence Framework Identifies Candidate Targets for Drug Repurposing in Alzheimer's Disease

Correspondences to: bingshan.li@vanderbilt.edu (B.L); Lang.Li@osumc.edu (L.L); chengf@ccf.org (F.C.)

Corresponding to Feixiong Cheng, Ph.D.
Lerner Research Institute, Cleveland Clinic
Tel: +1-216-444-7654; Fax: +1-216-636-0009
Email: chengf@ccf.org

Supplementary data include Supplementary Methods and Materials, 4 Supplementary Figures, 12 Supplementary Tables (10 excel files), and Supplementary References.

Supplementary Material and Methods

1. Filter of GWAS SNPs from large-scale studies

To exclude the redundant genetic signals indicated in different AD GWAS studies, we filtered the collected SNP list. Specifically, we first picked the SNP with the lowest *p* value from the collected SNPs, and then removed all the other SNPs within the 2 Mb region centered at the picked SNP; by this we removed SNPs that may represent the same underlying causal variants. For the remaining SNPs, we again picked the SNP with the lowest *p* value and repeated the process until there were no SNPs left. We used the selected SNPs to predict AD risk genes.

2. Integration of GWAS-derived closest genes to the risk loci

We obtained the nearest genes (MAPPED_GENE) for each SNP (**Table S1**) associated with AD from large-scale GWAS studies from GWAS Catalog. The nearest genes that were duplicate or not protein-coding genes were deleted. Finally, 108 GWAS-derived closest genes to the risk loci were retained (**Table S1**).

3. Construction of human protein-protein interactome

To build a comprehensive human protein-protein interactome, we assembled data from 15 common resources with various kinds of experimental evidences. Specifically, we focus on high-quality PPIs with five types of data.

(1) Binary PPIs tested by high-throughput yeast-two-hybrid (Y2H) systems: we combined binary PPIs tested from two public available high-quality Y2H datasets,[1, 2] and one *in-house* dataset;[3] (2) Kinase-substrate interactions by literature-derived low-throughput and high-throughput experiments from KinomeNetworkX,[4] Human Protein Resource Database (HPRD),[5] PhosphoNetworks,[6] PhosphositePlus,[7] DbPTM 3.0,[8] and Phospho. ELM.[9] (3) Carefully literature-curated PPIs identified by affinity purification followed by mass spectrometry (AP-MS), Y2H and by literature-derived low-throughput experiments, and protein three-dimensional structures from BioGRID,[10] PINA,[11] Instruct,[12] MINT,[13] IntAct,[14] and InnateDB.[15] (4) Signaling network by literature-derived low-throughput experiments as annotated in SignaLink2.0.[16] (5) Protein complexes data (56,000 candidate interactions) identified by a robust affinity purification-mass spectrometry methodology were collected from BioPlex V2.016.[17] The genes were mapped to their Entrez ID based on the NCBI database as well as their official gene symbols based on GeneCards (http://www.genecards.org/). Duplicated pairs were removed. Hence, inferred data, such as evolutionary analysis, gene expression data, and metabolic associations, were excluded. The resulting updated human interactome used here includes 351,444 protein-protein interactions (PPIs) connecting 17,706 unique proteins.

4. Collection of Alzheimer's disease-associated genes

In order to collect high-quality experimentally validated genes (AD seed

genes) for characterization of AD pathogenesis, we first manually curated experimentally validated (seed) genes in amyloidosis (amyloid) and tauopathy (tau). These genes satisfied at least one of the following criteria: i) gene validation in large-scale AD, amyloid or tauopathy GWAS studies.[18] The amyloid GWAS study refers to identifying genetic modifiers of amyloid-related traits, such as cerebrospinal fluid (CSF) levels of $A\beta_{42}$ and $A\beta_{42}$ burden measured by positron emission tomography (PET). The tauopathy GWAS study refers to identifying genetic modifiers of tau-related traits, such as cerebrospinal fluid (CSF) levels of tau or phosphorylated tau proteins, and neurofibrillary tangle (NFT); ii) *in vivo* experimental model evidence that knockdown or overexpression of the gene leads to AD-like amyloid or tau pathology. Based on these criteria, we obtained 54 seed genes related to amyloid and 27 seed genes related to tauopathy. We further integrated a list of AD seed genes via assembling multiple data sources as below: i) 54 amyloid seed genes and 27 tauopathy seed genes; ii) 47 late-onset AD common-risk genes identified by large-scale genetic studies; iii) 35 genes with at least two AD-causing mutations from the Human Gene Mutation Database (HGMD) [19](**Table S2)**; iv) 39 disease genes curated in at least 2 of 4 following disease gene databases: HGMD (n=63), DisGeNET (n=23, score≥0.2)[20], MalaCards (n=79)[21], and Open targets (n=81, overall score≥0.7 and literature score > 0)[22]. In summary, all AD seed genes are human genes via assembling experimental evidence either from human studies or AD mouse models. After

removing the duplicates, 144 AD seed genes were obtained (**Table S2)**.

5. Processing single cell/nucleus data and quality control.

The detailed data processing steps for each dataset (GSE98969, GSE140511, GSE147528, and GSE138852) are illustrated as below (**Table S3**).

GSE98969. The data used are from whole brain cells of 6 months 5XFAD (n = 16) and C57BL/6 (n = 16) mice that express CD45.[23] For quality control, cells with mitochondrial content >5% and UMIs < 500 were removed. Genes with mean expression smaller than 0.005 UMIs/cell were discarded for analysis. Data were normalized using a scaling factor of 10,000 and nUMI was regressed with a negative binomial model. Principle component analysis (PCA) was performed using the top 3000 most variable genes and clustering was performed using the top 38 PCAs and resolution of 0.6. After identifying clusters enriched in disease associated microglia (DAM) and homeostasis associated microglia (HAM) cells, differential expressed genes (DEGs) are compared between DAM and HAM by considering cells from 5XFAD mice only. The whole pipeline was completed with Seurat R package.

GSE140511. The data used are from nuclei of brains of 7 months 5XFAD (n = 3), Trem2 knock out 5XFAD (n = 3), Trem2 knock out C57BL/6 (n = 3), and wildtype C57BL/6 (n = 3) mice. The process for clustering different cell types

has been explained in the original literature already.[24] After that, we focused on microglia cells only, and reproduced the clustering procedures to isolates DAM and HAM cells. Considering all microglia cells, PCA was performed using the top 3000 most variable genes and sub-clustering was performed using the top 10 PCAs and resolution of 0.1. Again, after identifying clusters enriched in DAM and HAM cells, DEGs are compared between DAM and HAM by considering cells from 5XFAD mice only. The whole pipeline was completed with Seurat R package.

GSE147528. The data are generated from nuclei in both entorhinal cortex (EC) and superior frontal gyrus (SFG) regions from 10 frozen post-mortem human brain tissues. All 10 individuals are male and 3, 4, and 3 out of them are diagnosed with Braak stage 0, 2, and 6, respectively. For this dataset, we focused on astrocyte cells as its subtype named reactive astrocytes are associated with AD disease progress.[25] As indicated by the original literature, [25] cells from a given major type should recompute the size factor and highly variable genes. Therefore, by considering astrocyte cells only, clustering was first performed by *quickCluster* function and size factors were computed by *computeSumFactors* function with parameter min.mean = 0.1 in scran R package. Then count matrix was normalized by the computed size factors and log-transformed by function *logNormCounts* in scater R package. Top 1000 highly variable genes were selected by functions *modelGeneVar* and

getTopHVGs in scran R package. Functions *FindIntegrationAnchors* and *IntegrateData* in Seurat R package are used for batch effect correction, and clustering was performed using the top 12 PCAs and resolution of 0.2. After identifying clusters enriched in reactive astrocyte cells, DEGs are compared between reactive astrocytes and the rest of astrocytes cells for cells from EC and SFG regions, respectively.

GSE138852. The data used are from entorhinal cortex cells of 8 individuals AD (4) and control (4). The identification process of six cell types: microglia, astrocyte, neuron, oligodendrocytes, oligodendrocyte progenitor cells (OPCs), and endothelial cells has been explained in the original literature already.[26] The DEGs for each cell type are performed between AD and control individuals. The whole pipeline was completed with Seurat R package.

6. Identification of differentially expressed genes/proteins for enrichment analysis

Bulk RNA-seq. We collected 2 RNA-seq datasets from brain or brain microglial of 5XFAD mouse model from two studies.[27,28] The differential expression analysis was done with DESeq package in R.[29] The threshold for significance of differential expression was set to a conservative statistical threshold of False Discovery Rate (FDR) < 0.05 and fold change (FC) ≥ 1.2. After transferring to human-orthologous gene, we obtain two differentially expressed gene (DEG)

sets for 5XFAD_brain (n=17) and 5XFAD_microglial (n=432).

Furthermore, we collected 4 RNA-seq datasets from a recent study by Wang et al.[30] This study performed a genome-wide RNAseq to reveal the molecular mechanisms underlying microglia activation in response to pathological tau perturbation in Tg4510 mouse model. Microglia cells were isolated from Tg4510 brain and gene expression was profiled using RNA sequencing.[30] Four age groups of mice (2M, 4M, 6M, and 8M) were analyzed to capture longitudinal gene expression changes. Accession number for RNA-Seq data in Gene Expression Omnibus (GEO) is GSE123467. Given the larger number of DEGs identified by a threshold FC of 1.2, we used a stricter criterion (FDR< 0.05 and FC≥ 2) to determine DEGs compared with corresponding wide type mice. All DEGs identified in mice models were further mapped to unique human-orthologous genes using the Mouse Genome Informatics (MGI) database.[31] Finally, we obtained 4 DEG sets for Tg4510, including Tg4510_2M (788), Tg4510_4M (629), Tg4510_6M (1013) and Tg4510_8M (792).

Proteomics. The ten sets of DEPs were assembled from 3 AD transgenic models in two recent publications.[32,33] The first publication by Savas et al. performed global quantitative proteomic analysis in hAPP and hAPP-PS1 mouse models at young (3 month [M]) and old ages (12 M).[32] The samples were derived from frontal cortex (FC), hippocampal (HIP), and cerebellar (CB) extracts in mouse brain. The statistical significance of differential expression

of all proteins was assessed using a two-tailed one-sample t-test on their corresponding peptide quantification ratios between both conditions. The obtained *P* values were FDR-adjusted for multiple hypothesis testing using the Benjamini–Hochberg correction.[34] DEPs for each brain region in mouse were determined with the threshold of FDR < 0.05 and transferred to homologous human gene.[35] We obtained four sets of DEPs [hAPP_3M (n=363), hAPP_12M (n=624), hAPP-PS1_3M (n=262) and hAPP-PS1_12M (n=476)] after merging the DEPs from different brain regions.

The other publication by Kim et al. performed quantitative proteomics to uncover molecular and functional signatures in HIP of two transgenic mouse, including $ADLP_{APT}$ that carry three human transgenes (APP, PS1 and tau), and hAPP-PS1(5XFAD) mouse.[33] The $ADLP_{APT}$ mice, generated by crossing the 5XFAD strain with JNPL3 tau animals, could exhibit amyloid plaques, accelerated neurofibrillary tangle formation, neuronal loss in the CA1 area, and memory deficit at an early age. The 5XFAD mouse model develops early plaque formation, intraneuronal Aβ aggregation, neuron loss, and behavioral deficits[27]. Three different ages of mouse were used, including young (4 M), middle (7 M), and old (10 M). The statistical cut-off value for significance was set to *P*-value <0.05 for the Student's t-test and fold-change >1.25. After transferring to homologous human gene,[35] we obtained six sets of DEPs, including $ADLP_{APT}$_4M (n=53), $ADLP_{APT}$_7M (n=124), $ADLP_{APT}$_10M (n=299), hAPP-PS1_4M (n=54), hAPP-PS1_7M (n=168), and hAPP-PS1_10M (n=237).

7. Enrichment analysis

Differentially expressed gene/protein (DEG/DEP) sets from multiple data sources were collected for enrichment analysis. We performed enrichment analysis for network-predicted ARGs in AD using 16 DEG/DEP sets (**Table S7**) from bulk tissue gene expression and proteomics data. A list of 20,462 human protein-coding genes from the NCBI database were chosen as human genome background. Fisher's exact test was utilized to calculate the significant association (*P* value) between the 103 ARGs and the 16 input DEG/DEP sets.

8. Permutation tests for LCC

A permutation test, also called a randomization test, is a standard test to compute statistical significance. We extracted 103 (the same number with ARGs) randomly selected genes with similar connectivity (degree) as the original ARG genes in the human interactome. Then we calculated the largest connected component [LCC] for this randomly gene set. We performed the permutation testing as below:

$$P = \frac{\#\{S_m(p) > S_m\}}{\#\{total\ permutations\}} \qquad \text{(S1)}$$

A nominal *P* was computed by counting the number of permutations (S_m (p)) greater than the number of observed LCC (Sm). Then we performed 1,000 times permutations to calculate the statistical significance.

Supplemental figure titles and legends

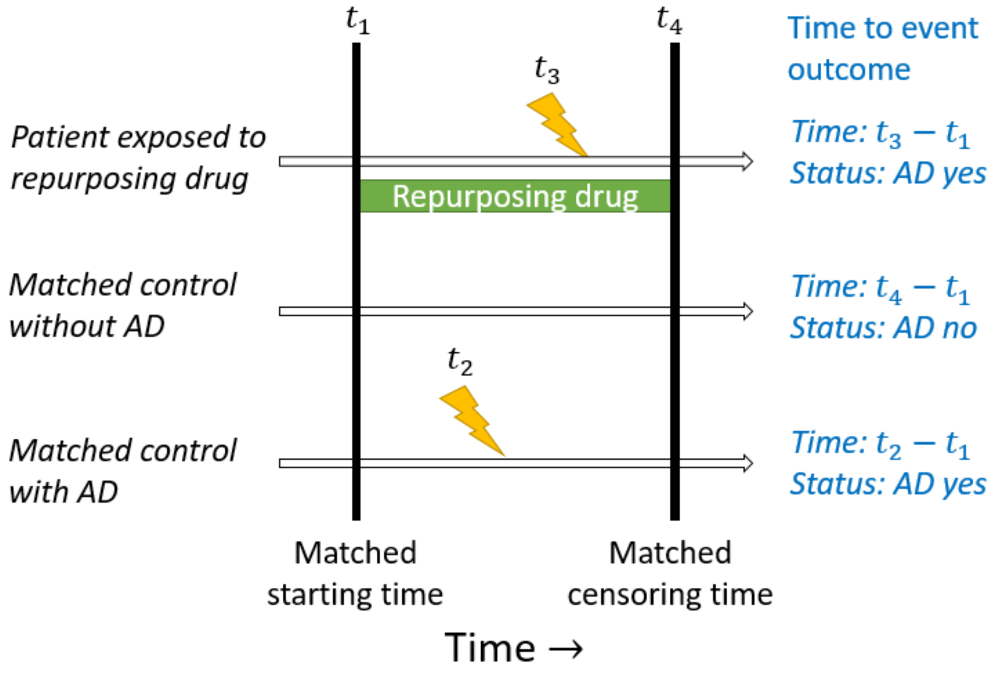

Figure S1. Matched control for repurposing drug. More details for study design are provided in the Methods section.

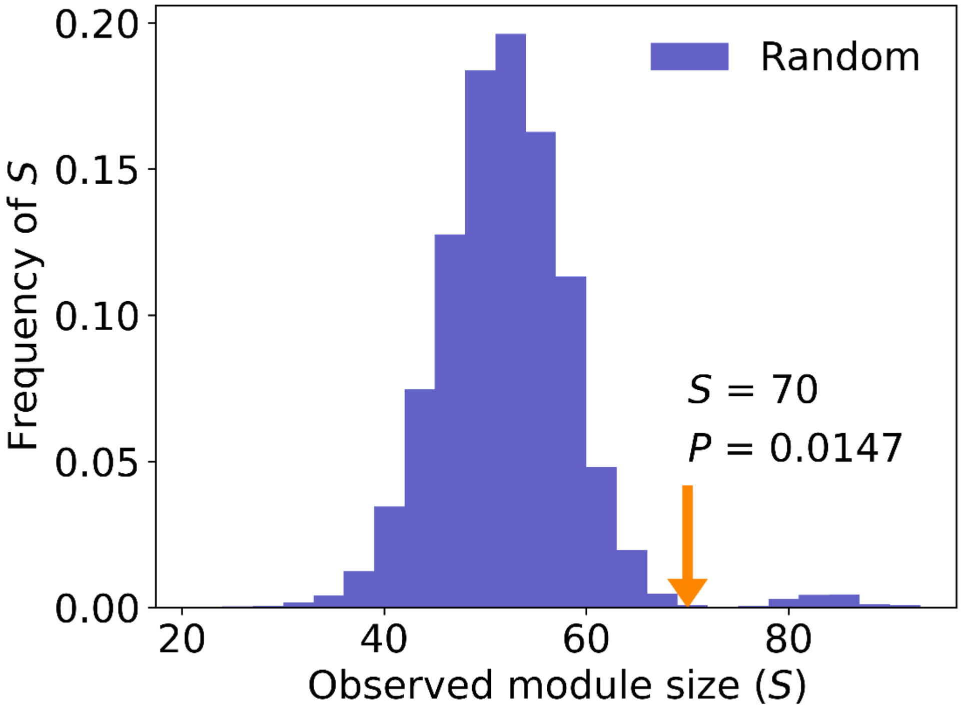

Figure S2. The largest connected component (LCC) analysis for Alzheimer's disease module. High-confidence AD risk genes (ARGs) form significantly disease module in the human interactome (P = 0.0147, 10,000 permutation test). ARGs products (proteins) are likely to cluster in the same network neighborhood or disease module within the human protein-protein interactome. The observed module sizes (*S*), 70 (seed genes, orange line) are significantly larger than the random expectation.

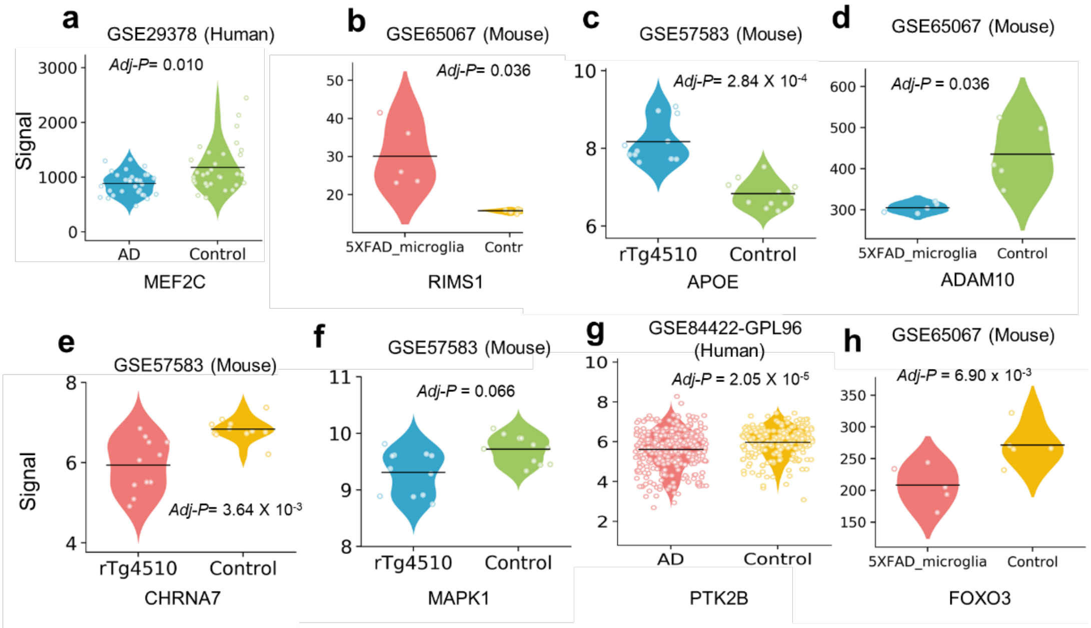

Figure S3. Differential gene expression of AD risk genes (ARGs) between AD and controls. The genes include MEF2C (**a**), RIMS1 (**b**), APOE (**c**), ADAM10 (**d**), CHRNA7 (**e**), MAPK1 (**f**), PTK2B (**g**), and FOXO3 (**h**). The signal value of AD (patient or mouse model) and controls were extracted from original microarray datasets in Gene Expression Omnibus (GEO) database. *P* value was computed for each gene between AD and controls using one side Wilcoxon test. Adjusted *P* value (*adj-P*) was calculated based on the Benjamini−Hochberg approach.

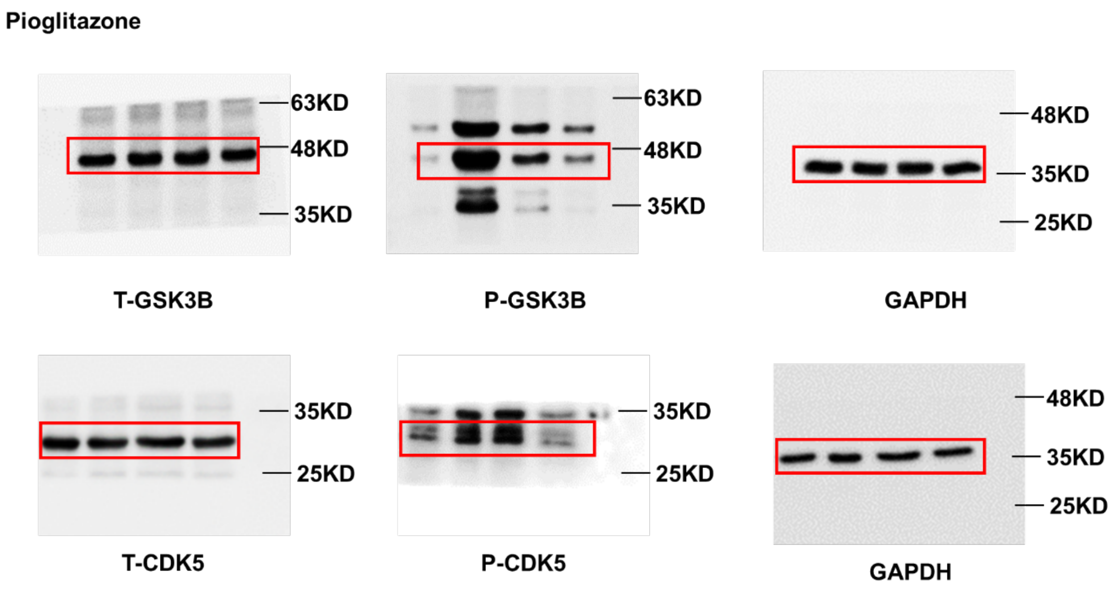

Figure S4. Effects of pioglitazone on LPS-induced activation of GSK3β and CDK5 in human microglia HMC3 cells. HMC3 cells were pretreated with sildenafil and followed LPS treatment (1 μg/mL, 30 min). The total cell lysates were collected and subjected to Western blot analysis. Quantification data represent mean ± s.d. of two independent experiments.

Supplemental table titles and legends

Table S1. Summary of 15 large-scale GWAS studies with AD in diverse population groups, conducted between 2007 and 2019 (.xls).

Table S2. The list of AD-associated genes from the Open Targets database as well as AD seed genes with experimental evidence (.xls).

Table S3. Summary of sc/snRNA-Seq datasets and microarray datasets used in this study (.xls).

Table S4. The detailed information of 15,367 drug–target interactions connecting 1,608 FDA-approved drugs and 2,251 unique human targets (.xls).

Table S5. Phenotype definitions by ICD9/10 codes.

Table S6. The list of high-confidence AD risk genes (ARGs) predicted by Bayesian model selection method (.xls).

Table S7. Summary of bulk RNA-seq data and proteomics dataset from AD transgenic mouse models (.xls).

Table S8. The detailed information of AD multi-omics evidence for 103 predicted ARGs (.xls).

Table S9. The result of biological pathway enrichment analysis using 37 likely causal genes (.xls).

Table S10. The list of typical ARGs from statistically significant biological pathways and related AD literature evidence (.xls).

Table S11. The list of 130 predicted drugs with reported anti-AD clinical, in vitro/in vivo data (.xls).

Table S12. Comparison of largest connected component [LCC] analysis between the nearest genes to the risk loci and ARGs

Table S5: phenotype definitions by ICD9/10 codes.

**Alzheimer's disease** [36; 37]
3310, F00, F000A, F001A, F002A, F009A, G30, G300, G301, G308, G309
**Type 2 diabetes** [38; 39]
25000, 25050, 25002, 25052, 25010, 25060, 25012, 25062, 25020, 25070, 25022, 25072, 25030, 25080, 25032, 25082, 25040, 25090, 25042, 25092, E089, E1100, E1101, E1110, E1121, E1122, E1129, E11311, E11319, E113219, E113291, E113292, E113293, E113299, E113319, E113391, E113392, E113393, E113399, E113419, E113491, E113492, E113493, E113499, E113519, E113591, E113592, E113593, E113599, E1136, E1139, E1140, E1142, E1143, E1144, E1149, E1151, E1152, E1159, E11610, E11618, E11620, E11621, E11628, E11630, E11641, E11649, E1165, E1169, E118, E119, E1300, E1310, E1311, E1322, E1329, E1339, E1349, E1351, E13628, E1365 , E1369 , E138 , E139
**Hypertension** [40; 41]
4010, 4011, 4019, I10, I169
**Coronary artery disease**[40; 42; 43]
410, 411, 412, 4131 414, I20, I21, I22, I23, I24, I25

Table S12 Comparison of largest connected component [LCC] analysis between the nearest genes to the risk loci and ARGs.

Gene set	# gene	# human interactome	LCC	p-value
Nearest gene to the risk loci	108	96	7	0.217
High-confidence AD risk genes (ARGs)	103	103	70	0.015

Supplementary References

1. Rolland, T., Tasan, M., Charloteaux, B., Pevzner, S.J., Zhong, Q., Sahni, N., Yi, S., Lemmens, I., Fontanillo, C., Mosca, R., et al. (2014). A proteome-scale map of the human interactome network. Cell 159, 1212-1226.

2. Rual, J.F., Venkatesan, K., Hao, T., Hirozane-Kishikawa, T., Dricot, A., Li, N., Berriz, G.F., Gibbons, F.D., Dreze, M., Ayivi-Guedehoussou, N., et al. (2005). Towards a proteome-scale map of the human protein-protein interaction network. Nature 437, 1173-1178.

3. Cheng, F., Desai, R.J., Handy, D.E., Wang, R., Schneeweiss, S., Barabási, A.L., and Loscalzo, J. (2018). Network-based approach to prediction and population-based validation of in silico drug repurposing. Nature communications 9, 2691.

4. Cheng, F., Jia, P., Wang, Q., and Zhao, Z. (2014). Quantitative network mapping of the human kinome interactome reveals new clues for rational kinase inhibitor discovery and individualized cancer therapy. Oncotarget 5, 3697-3710.

5. Keshava Prasad, T.S., Goel, R., Kandasamy, K., Keerthikumar, S., Kumar, S., Mathivanan, S., Telikicherla, D., Raju, R., Shafreen, B., Venugopal, A., et al. (2009). Human Protein Reference Database--2009 update. Nucleic acids research 37, D767-772.

6. Hu, J., Rho, H.S., Newman, R.H., Zhang, J., Zhu, H., and Qian, J. (2014). PhosphoNetworks: a database for human phosphorylation networks. Bioinformatics (Oxford, England) 30, 141-142.

7. Hornbeck, P.V., Zhang, B., Murray, B., Kornhauser, J.M., Latham, V., and Skrzypek, E. (2015). PhosphoSitePlus, 2014: mutations, PTMs and recalibrations. Nucleic acids research 43, D512-520.

8. Lu, C.T., Huang, K.Y., Su, M.G., Lee, T.Y., Bretana, N.A., Chang, W.C., Chen, Y.J., Chen, Y.J., and Huang, H.D. (2013). DbPTM 3.0: an informative resource for investigating substrate site specificity and functional association of protein post-translational modifications. Nucleic acids research 41, D295-305.

9. Dinkel, H., Chica, C., Via, A., Gould, C.M., Jensen, L.J., Gibson, T.J., and Diella, F. (2011). Phospho.ELM: a database of phosphorylation sites--update 2011. Nucleic acids research 39, D261-267.
10. Oughtred, R., Stark, C., Breitkreutz, B.J., Rust, J., Boucher, L., Chang, C., Kolas, N., O'Donnell, L., Leung, G., McAdam, R., et al. (2019). The BioGRID interaction database: 2019 update. Nucleic acids research 47, D529-d541.
11. Cowley, M.J., Pinese, M., Kassahn, K.S., Waddell, N., Pearson, J.V., Grimmond, S.M., Biankin, A.V., Hautaniemi, S., and Wu, J. (2012). PINA v2.0: mining interactome modules. Nucleic acids research 40, D862-865.
12. Meyer, M.J., Das, J., Wang, X., and Yu, H. (2013). INstruct: a database of high-quality 3D structurally resolved protein interactome networks. Bioinformatics (Oxford, England) 29, 1577-1579.
13. Licata, L., Briganti, L., Peluso, D., Perfetto, L., Iannuccelli, M., Galeota, E., Sacco, F., Palma, A., Nardozza, A.P., Santonico, E., et al. (2012). MINT, the molecular interaction database: 2012 update. Nucleic acids research 40, D857-861.
14. Orchard, S., Ammari, M., Aranda, B., Breuza, L., Briganti, L., Broackes-Carter, F., Campbell, N.H., Chavali, G., Chen, C., del-Toro, N., et al. (2014). The MIntAct project--IntAct as a common curation platform for 11 molecular interaction databases. Nucleic acids research 42, D358-363.
15. Breuer, K., Foroushani, A.K., Laird, M.R., Chen, C., Sribnaia, A., Lo, R., Winsor, G.L., Hancock, R.E., Brinkman, F.S., and Lynn, D.J. (2013). InnateDB: systems biology of innate immunity and beyond--recent updates and continuing curation. Nucleic acids research 41, D1228-1233.
16. Csabai, L., Olbei, M., Budd, A., Korcsmaros, T., and Fazekas, D. (2018). SignaLink: Multilayered Regulatory Networks. Methods in molecular biology (Clifton, NJ) 1819, 53-73.
17. Huttlin, E.L., Ting, L., Bruckner, R.J., Gebreab, F., Gygi, M.P., Szpyt, J., Tam, S., Zarraga, G., Colby, G., Baltier, K., et al. (2015). The BioPlex Network: A

Systematic Exploration of the Human Interactome. Cell 162, 425-440.
18. Deming, Y., Li, Z., Kapoor, M., Harari, O., Del-Aguila, J.L., Black, K., Carrell, D., Cai, Y., Fernandez, M.V., Budde, J., et al. (2017). Genome-wide association study identifies four novel loci associated with Alzheimer's endophenotypes and disease modifiers. Acta neuropathologica 133, 839-856.
19. Stenson, P.D., Mort, M., Ball, E.V., Evans, K., Hayden, M., Heywood, S., Hussain, M., Phillips, A.D., and Cooper, D.N. (2017). The Human Gene Mutation Database: towards a comprehensive repository of inherited mutation data for medical research, genetic diagnosis and next-generation sequencing studies. Human genetics 136, 665-677.
20. Pinero, J., Bravo, A., Queralt-Rosinach, N., Gutierrez-Sacristan, A., Deu-Pons, J., Centeno, E., Garcia-Garcia, J., Sanz, F., and Furlong, L.I. (2017). DisGeNET: a comprehensive platform integrating information on human disease-associated genes and variants. Nucleic acids research 45, D833-d839.
21. Rappaport, N., Twik, M., Plaschkes, I., Nudel, R., Iny Stein, T., Levitt, J., Gershoni, M., Morrey, C.P., Safran, M., and Lancet, D. (2017). MalaCards: an amalgamated human disease compendium with diverse clinical and genetic annotation and structured search. Nucleic acids research 45, D877-d887.
22. Koscielny, G., An, P., Carvalho-Silva, D., Cham, J.A., Fumis, L., Gasparyan, R., Hasan, S., Karamanis, N., Maguire, M., Papa, E., et al. (2017). Open Targets: a platform for therapeutic target identification and validation. Nucleic acids research 45, D985-d994.
23. Keren-Shaul, H., Spinrad, A., Weiner, A., Matcovitch-Natan, O., Dvir-Szternfeld, R., Ulland, T.K., David, E., Baruch, K., Lara-Astaiso, D., Toth, B., et al. (2017). A Unique Microglia Type Associated with Restricting Development of Alzheimer's Disease. Cell 169.
24. Zhou, Y., Song, W.M., Andhey, P.S., Swain, A., Levy, T., Miller, K.R., Poliani, P.L., Cominelli, M., Grover, S., Gilfillan, S., et al. (2020). Human and mouse single-nucleus transcriptomics reveal TREM2-dependent and TREM2-

independent cellular responses in Alzheimer's disease. Nat Med 26, 131-142.

25. Leng, K., Li, E., Eser, R., Piergies, A., Sit, R., and Tan, M., et al. (2020). Molecular characterization of selectively vulnerable neurons in Alzheimer’s Disease. Nat Neurosci. 2021 Feb;24(2):276-287.

26. Grubman, A., Chew, G., Ouyang, J.F., Sun, G., Choo, X.Y., McLean, C., Simmons, R.K., Buckberry, S., Vargas-Landin, D.B., Poppe, D., et al. (2019). A single-cell atlas of entorhinal cortex from individuals with Alzheimer's disease reveals cell-type-specific gene expression regulation. Nat Neurosci 22, 2087-2097.

27. Bouter, Y., Kacprowski, T., Weissmann, R., Dietrich, K., Borgers, H., Brauss, A., Sperling, C., Wirths, O., Albrecht, M., Jensen, L.R., et al. (2014). Deciphering the molecular profile of plaques, memory decline and neuron loss in two mouse models for Alzheimer's disease by deep sequencing. Frontiers in aging neuroscience 6, 75.

28. Grubman, A., Choo, X.Y., Chew, G., Ouyang, J.F., Sun, G., Croft, N.P., Rossello, F.J., Simmons, R., Buckberry, S., and Landin, D.V. (2019). Mouse and human microglial phenotypes in Alzheimer's disease are controlled by amyloid plaque phagocytosis through Hif1α. bioRxiv, 639054.

29. Anders, S. (2010). Analysing RNA-Seq data with the DESeq package. Mol Biol 43, 1-17.

30. Wang, H., Li, Y., Ryder, J.W., Hole, J.T., Ebert, P.J., Airey, D.C., Qian, H.-R., Logsdon, B., Fisher, A., and Ahmed, Z. (2018). Genome-wide RNAseq study of the molecular mechanisms underlying microglia activation in response to pathological tau perturbation in the rTg4510 tau transgenic animal model. Molecular neurodegeneration 13, 65.

31. Eppig, J.T., Smith, C.L., Blake, J.A., Ringwald, M., Kadin, J.A., Richardson, J.E., and Bult, C.J. (2017). Mouse Genome Informatics (MGI): resources for mining mouse genetic, genomic, and biological data in support of primary and translational research. In Systems Genetics. (Springer), pp 47-73.

32. Savas, J.N., Wang, Y.Z., DeNardo, L.A., Martinez-Bartolome, S., McClatchy, D.B., Hark, T.J., Shanks, N.F., Cozzolino, K.A., Lavallee-Adam, M., Smukowski, S.N., et al. (2017). Amyloid Accumulation Drives Proteome-wide Alterations in Mouse Models of Alzheimer's Disease-like Pathology. Cell Reports 21, 2614-2627.

33. Kim, D.K., Park, J., Han, D., Yang, J., Kim, A., Woo, J., Kim, Y., and Mook-Jung, I. (2018). Molecular and functional signatures in a novel Alzheimer's disease mouse model assessed by quantitative proteomics. Molecular neurodegeneration 13, 2.

34. Benjamini, Y., and Hochberg, Y. (1995). Controlling the false discovery rate: a practical and powerful approach to multiple testing. Journal of the Royal statistical society: series B (Methodological) 57, 289-300.

35. Eppig, J.T., Smith, C.L., Blake, J.A., Ringwald, M., Kadin, J.A., Richardson, J.E., and Bult, C.J. (2017). Mouse Genome Informatics (MGI): Resources for Mining Mouse Genetic, Genomic, and Biological Data in Support of Primary and Translational Research. Methods in molecular biology (Clifton, NJ) 1488, 47-73.

36. Wei, W.Q., Teixeira, P.L., Mo, H., Cronin, R.M., Warner, J.L., and Denny, J.C. (2016). Combining billing codes, clinical notes, and medications from electronic health records provides superior phenotyping performance. Journal of the American Medical Informatics Association (JAMIA), 23, e20-27.

37. Wilkinson, T., Ly, A., Schnier, C., Rannikmae, K., Bush, K., Brayne, C., Quinn, T.J., and Sudlow, C.L.M. (2018). Identifying dementia cases with routinely collected health data: A systematic review. Alzheimer's & dementia : the journal of the Alzheimer's Association 14, 1038-1051.

38. Kho, A.N., Hayes, M.G., Rasmussen-Torvik, L., Pacheco, J.A., Thompson, W.K., Armstrong, L.L., Denny, J.C., Peissig, P.L., Miller, A.W., Wei, W.Q., et al. (2012). Use of diverse electronic medical record systems to identify genetic risk for type 2 diabetes within a genome-wide association study. Journal of the

American Medical Informatics Association (JAMIA), 19, 212-218.
39. Wei, W.Q., Leibson, C.L., Ransom, J.E., Kho, A.N., Caraballo, P.J., Chai, H.S., Yawn, B.P., Pacheco, J.A., and Chute, C.G. (2012). Impact of data fragmentation across healthcare centers on the accuracy of a high-throughput clinical phenotyping algorithm for specifying subjects with type 2 diabetes mellitus. Journal of the American Medical Informatics Association (JAMIA), 19, 219-224.
40. Federman, D.G., Krishnamurthy, R., Kancir, S., Goulet, J., and Justice, A. (2005). Relationship between provider type and the attainment of treatment goals in primary care. The American journal of managed care 11, 561-566.
41. Banerjee, D., Chung, S., Wong, E.C., Wang, E.J., Stafford, R.S., and Palaniappan, L.P. (2012). Underdiagnosis of hypertension using electronic health records. American journal of hypertension 25, 97-102.
42. Smilowitz, N.R., Oberweis, B.S., Nukala, S., Rosenberg, A., Zhao, S., Xu, J., Stuchin, S., Iorio, R., Errico, T., Radford, M.J., et al. (2016). Association Between Anemia, Bleeding, and Transfusion with Long-term Mortality Following Noncardiac Surgery. The American journal of medicine 129, 315-323.e312.
43. Abul-Husn, N.S., Manickam, K., Jones, L.K., Wright, E.A., Hartzel, D.N., Gonzaga-Jauregui, C., O'Dushlaine, C., Leader, J.B., Lester Kirchner, H., Lindbuchler, D.M., et al. (2016). Genetic identification of familial hypercholesterolemia within a single U.S. health care system. Science (New York, NY) 354.
